# Visualization-Based Discovery of Vanin-1 Inhibitors for Colitis

**DOI:** 10.3389/fchem.2021.809495

**Published:** 2022-01-28

**Authors:** Guankai Wang, Jingjing Wang, Lupei Du, Minyong Li

**Affiliations:** Key Laboratory of Chemical Biology (MOE), Department of Medicinal Chemistry, School of Pharmaceutical Sciences, Cheeloo College of Medicine, Shandong University, Jinan, China

**Keywords:** vanin-1, VNN1 inhibitors, probe, visualization of biological activity, colitis model in mice

## Abstract

The main effect of Vanin-1/VNN1 is related to its pantetheinase sulfhydrylase activity, which can hydrolyze pantetheine into pantothenic acid and cysteamine. In recent studies, the enzymatic activity of vanin-1/VNN1 has been found to be essential in the development of many diseases. The study of specific vanin-1/VNN1 inhibitors can give us a deeper understanding of its role in the disease process. In this study, different skeletal inhibitors were designed and synthesized using pyrimidine amide compounds as lead compounds. In order to screen inhibitors intuitively, a fluorescent probe PA-AFC for *in vitro* evaluation of inhibitors was designed and synthesized in this study, which has good sensitivity and specificity. The bioluminescent probe PA-AL was then used for cellular level and *in vivo* inhibitor evaluation. This screening method was convenient, economical and highly accurate. Finally, these inhibitors were applied to a mouse colitis model, confirming that vanin-1 is useful in IBD and providing a new therapeutic direction.

**GRAPHICAL ABSTRACT:**
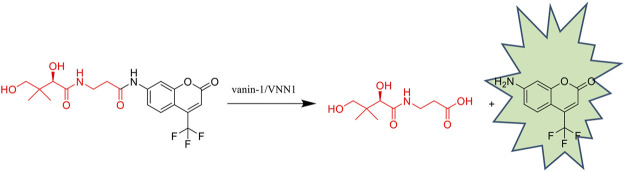


## Introduction

Vanin-1 is expressed on the surface of thymic stromal cell membrane around blood vessels, which is anchored by glycosylphosphatidylinositol (GPI) (Rommelaere et al., 2013). The main effect of Vanin-1/VNN1 is related to its pantetheinase sulfhydrylase activity, which can hydrolyze pantetheine into pantothenic acid and cysteamine (Dupre et al., 1970). Pantothenic acid is also known as vitamin B5, which can be used as a precursor of coenzyme A (Naquet et al., 2014). The vanin-1/VNN1-cysteamine pathway is involved in pro-inflammatory and oxidative stress under physiological conditions (Boersma et al., 2014). In recent years, many studies in both human and animal models have confirmed Vanin-1 as a key player in disease progression. For example, it is reported that 2,4,6-trinitrobenzenesulfonic acid (TNBS)-induced mouse colitis model, the lack of vanin-1 gene can protect mice from colitis (Berruyer et al., 2006). Similarly, vanin-1 specific inhibitors have also become an important tool for studying the biological effects of vanin-1. It may not only provide new strategies for disease treatment, but also has the potential to develop new therapeutic anti-inflammatory drugs.

There are few studies on vanilloid-1 specific inhibitors in the world and they are mainly in the form of patents. In 2013, Schalkwijk et al. developed a ubiquine analogue inhibitor, of which the most active compound is RR6 (IC_50_ = 0.54 μM). This compound can competitively and reversibly inhibit pantothenate activity, and its efficacy has been confirmed in an *in vivo* rat model. So far, RR6 has been widely used in various studies to analyze the role of vanin-1 targets in various disease processes (Jansen et al., 2013; van Diepen et al., 2014; Wedel et al., 2016).

From 2016 to 2019, several series of compound patents with different core types were reported as vanin-1 specific inhibitors. The number of compounds is not only huge, but the IC_50_ values have reached the nanomolar level. Pfizer applied for two patents in 2016 and 2018. In 2016, the vanin-1 enzyme inhibitor patent is a new heterocyclic compound as an inhibitor of vanin-1 enzyme. On this basis, in 2018 the company refined the skeleton for pyrimidine amides. In 2018, Shen et al. applied for a patent for heteropolyester compounds as vanin-1 inhibitors, while Burch et al. applied for a patent for aminothiazole compounds in 2019. We used pyrimidine amide compounds as lead compounds to change the skeleton structure, hoping to discover a new skeleton for inhibitors.

Recently, some probes were designed for the detection of vanin-1 enzyme activity, and reflecting the inhibitory effect of its inhibitors, measure their IC_50_ value, so that they can achieve the purpose of visually screening inhibitors (Gurung et al., 2020). Compared with traditional detection methods, depending on the connected luminophore, the scope of application becomes wider and wider. In 2010, Ruan et al. linked pantothenic acid with 4-amino-methylcoumarin fluorophore to synthesize the probe AMC-Pan (Ruan et al., 2010). The probe can not only be used for the detection of vanin-1 enzyme activity, but also can be used to screen inhibitors. But this probe is not suitable for cell, deep tissue imaging, and small animal live imaging. In 2017, Ma’s group developed a ratiometric fluorescent probe CV-PA (Hu et al., 2017), which was successfully applied to fluorescence imaging of living cells, but the probe has not been applied to *in vivo* detection. In 2018, the Li group developed the bioluminescence probe, PA-AL (Lin et al., 2018). Its emission wavelength is 590 nm, which is the first probe that can be used in the detection of vanin-1 enzyme *in vivo* (Ueno et al., 2004). PA-AL can effectively reflect the endogenous vanin-1 level and realize dynamic real-time monitoring. Because of its excellent selectivity and sensitivity, PA-AL will serve as an important tool *in vivo* for studying the effects of vanin-1 enzyme and visually screening inhibitors.

## Experimental Section

### Synthesis

Synthesis of probe PA-AFC and inhibitors were completed within a few steps as depicted in Supplementary Scheme S1–S3 of the Supporting Information (SI). The detailed synthesis is described in the SI.

The synthesis of probe **PA-AFC** mainly goes through three steps. The first step is to use the acetal structure to condense the two hydroxyl groups in the pantothenic acid structure to protect it, and only expose the carboxyl group to avoid the influence on the reaction. The second step uses isobutyl chloroformate and N-methylmorpholine to condense the carboxyl group of pantothenic acid with the amino group of the fluorophore AFC to form an amide structure. The third step uses acetic acid to remove the acetal protecting group.

The synthesis of **compounds a-h** first utilizes the nucleophilic addition of aldehyde groups and amino groups under acidic conditions to generate imine structures. The imine is unstable, so use sodium cyanoborohydride or sodium borohydride to directly reduce it to a secondary amine structure. The ester group is then used to hydrolyze under alkaline conditions to generate its sodium salt, and hydrochloric acid is used to adjust the pH to neutral or make it generate carboxylic acid. Finally, the condensing agent HATU is used to condense the carboxylic acid and the amino group in the spiroalkane to form an amide structure.

The synthesis of **compound i-j** first involves the nucleophilic substitution reaction of amino and halogen using a palladium catalyst and its ligand under alkaline conditions. Then the ester group is hydrolyzed under alkaline conditions to generate its sodium salt, and the pH is adjusted to neutral with hydrochloric acid. Finally, SOCl_2_ is used to make it an acid chloride, and the secondary amine in the spiroalkane is nucleophilically attacked to form an amide.

All compounds have been confirmed by characterization methods, and their purity is >95%.

### Sensitivity Determination of Fluorescent Probe PA-AFC

After the fluorescent probe PA-AFC reacts, the fluorophore AFC can be obtained, and the light signal can be collected, so the change of fluorescence intensity can reflect the sensitivity of the probe.

In this study, the probe response to different concentrations of human recombinant VNN1 enzyme was measured to study the sensitivity of the fluorescent probe PA-AFC. Human recombinant VNN1 solutions of different concentrations were prepared with buffer solution (500, 250, 125, 62.5, 31.25, 15.63, 7.81, 3.91, 1.95, 0.98, and 0.49 ng/ml). Prepare the fluorescent probe PA-AFC solution with a buffer solution at a concentration of 20 μM. Add 50 μl of the above-mentioned VNN1 solution to a black 96-well plate, then add 50 μl of probe solution to each well, and immediately transfer to a 37°C shaker for 30 min. They were placed in a POLARstar Omega microplate reader, a 420 nm filter for excitation light, a 510 nm filter for emission light, and record the fluorescence intensity data at 510 nm.

### Selective Determination of Fluorescent Probe PA-AFC

To prove that the detected fluorescent signal is generated by the reaction between human recombinant VNN1 enzyme and probe PA-AFC, this study incubated the probe with several interfering substances, and the fluorescence intensity of the probe allowed the selectivity of the probe to be determine.

In this study, the interfering substances FeCl_3_, MgCl_2_, BaCl_2_, MnSO_4_, CaCl_2_, ZnCl_2_, AlCl_3_, PbCl_2_, and CuSO_4_ were prepared with a buffer solution to a concentration of 1 mM, and diluted GSH, D-cysteine, glucose, VcNa, L-valine, and Hcy to 1 mM, APN and GGT were diluted to a concentration of 50 U/L, and TYR was diluted to a concentration of 100 ng/ml. Dilute the recombinant human VNN1 enzyme concentration to 20 ng/ml, and dilute the probe PA-AFC to 20 μM with buffer.

Take 50 μl of probe solution and 50 μl of different substance solutions and mix them in a black 96-well plate, and incubate at 37°C for 30 min in a constant temperature shaker. Immediately after taking it out, place it in a POLARstar Omega microplate reader, use a 420 nm filter for excitation light, select a 510 nm filter for emission light, record the fluorescence intensity data at 510 nm and emission spectrum.

Meanwhile, Fe^3+^ is an efficient emission quencher for most fluorescent indicators. To further confirm the fluorescence quenching of probe PA-AFC by Fe^3+^, fluorescence microscopic imaging experiment and fluorescence quantum yield experiment were conducted (Sahoo and Crisponi, 2019).

Dilute the probe PA-AFC to 20 μM with buffer, and dilute the Fe^3+^ to 1 mM with buffer. ES-2-Fluc cells were plated on glass-bottom culture dishes and incubated at 37°C for 12 h. Add 500 μl fluorescent probe solution to each well, add 500 μl PBS buffer in the control group, and add 500 μl PBS buffer solution containing Fe^3+^ in the experimental group. After mixing, place the small dish plate on a shaker at 37°C and incubate for 30 min. Take the small dish plate out of the shaker and place it inverted fluorescence microscope for imaging.

Accordance with ultraviolet-visible spectrophotometer absorbance value of compound (between 0.01 and 0.05), and then determined by fluorescence spectrophotometer maximum excitation wavelength of fluorescence spectrum peak area, after the abbe refractometer is used to test the same conditions of different solvent refractive index data from generation into the following formula to calculate quantity of fluorescent quantum yield of the fluorescent samples under test.

$$\Phi_{X}=\Phi_{S}\left(A_{S}/A_{X}\right)\left(F_{X}/F_{S}\right){\left(\eta_{X}/\eta_{S}\right)}^{2}$$

In the formula, φ and A respectively refer to the quantum yield and the absorbance at the excitation wavelength, ƞ and F respectively represent the refractive index of the solvent and the peak area of the fluorescence emission spectrum, X and S respectively are the substance to be measured and the reference substance. Here, fluorescein is selected as the reference substance, and its quantum yield in 1 N NaOH aqueous solution is 92%.

Similarly, in order to evaluate the merits and disadvantages of our probes, we comprehensively compared the published fluorescent probes, and summarized them as depicted in Table 1.

**TABLE 1:** The summary of fluorescent probes for VNN1.

Probe	Advantage	Disadvantage
Ruan et al reported probe Pantothenate-AMC (Ruan et al., 2010)	Quantitative determination of vanin-1 activity in cells and tissues	Its excitation wavelength (340 nm) is very short, easy to confuse *in vivo* bioluminescence, is not conducive to cell imaging
Hu et al reported probe CV-PA (Hu et al., 2017)	It showed a linear (I_628_/_582_ nm) fluorescence response to vanin-1 in the range of 5–400 ng/ml with a detection limit of 4.7 ng/ml	It has not been successfully used for vanin-1 detection *in vivo*, and the distance between the two emission centers (46 nm) is too close to obtain a single fluorescence channel clearly
Qian et al reported fluorescent probe TMN-PA (Qian et al., 2020)	Prominent cell membrane permeability, large Stokes shift, good specificity, low toxicity, and an excellent NIR emission (658 nm)	The maximum emission wavelength is 645 nm
Qian et al reported probe DCM-PA (Qian et al., 2021)	DCM-PA has short response time (30 min), high selectivity and low sensitivity (DL = 0.69 ng/ml)	The maximum emission wavelength is 640 nm
Yang et al reported probe CYLP (Yang et al., 2020)	detection limit of 0.02 ng/ml, CYLP exhibited outstanding selectivity and sensitivity toward pantetheinase with NIR emission wavelength (>700 nm)	
Lu et al reported probe Cy-Pa (Lu et al., 2021)	quantitatively and qualitatively detected vanin-1 concentrations in HepG2 and HepG2/DDP cells or tumor tissues of tumor-bearing mice	
PA-AFC	The fluorescence intensity I_510_/I_420_ nm had a good linear relationship with vanin-1 concentration in the range of 0–500 ng/ml (*R* ^2^ = 0.9975)	Excitation wavelength (338 nm) is very short, convenient to cell imaging

### Determination of the Inhibitory Activity Against Human Recombinant VNN1 Enzyme *in vitro*

This study was used human recombinant VNN1 enzyme and fluorescent probe PA-AFC to determine the IC_50_ value of inhibitors.

Dilute the lead compound and each inhibitor to be tested with buffer to, for example, 400, 200, 100, 50, 25, 12.50, 6.25, 3.13, and 1.56 μM, 781 nM, 391 nM The gradient concentration of the solution. Prepare the human recombinant VNN1 enzyme solution at a concentration of 30 ng/ml. Prepare the fluorescent probe PA-AFC solution with a concentration of 20 μM.

Add 50 μl of gradient concentration of the inhibitor to be tested to each well of the black 96-well plate, and then continue to add 50 μl of the newly prepared human recombinant VNN1 enzyme solution to each well. Among them, the control group and the blank group were replaced with the same volume of buffer. After the solution is mixed, place the black 96-well plate on a shaker at 37°C for 30 min. Continue to add 100 μl of the prepared fluorescent probe solution to each well of the black 96-well plate. After mixing, place the black 96-well plate on a shaker at 37°C and incubate for 30 min. Take the black 96-well plate out of the shaker and place it in a POLARstar Omega microplate reader. Use a 420 nm filter for the excitation light and a 510 nm filter for the emission light. Record the fluorescence intensity data at 510 nm. Through GraphpadPrism mapping, the IC_50_ value of each inhibitor to be tested was obtained.

### Cytotoxicity Test

The study chose the MTT method to study the toxicity of inhibitors to human ovarian cancer cells ES-2-Fluc expressing firefly luciferase. Dilute the cells to a cell suspension with a concentration of 8 × 10^4^ cells/ml, take a completely transparent 96-well plate, add 100 μl to each well to reach about 8,000 cells in each well, and incubate at 37°C for 12 h. Dilute the lead compound and each inhibitor to be tested to the final concentration of 250, 125, and 62.5 μM. Add 100 μl to each well of a 96-well plate and incubate at 37°C for 12 h. Pipette 20 μl of 0.5 mg/ml MTT solution into each well of a 96-well plate, and incubate at 37°C for 4 h. Add 150 μl of DMSO to each well, shake at 22°C for 5 min and mix, place in a POLARstar Omega microplate reader, read the OD value of each well at 570 nm, and calculate the cell survival rate.

### Inhibitory Activity of Vanin-1 Enzyme at the Cellular Level

This study explores the inhibition of Vanin-1 enzyme at the cellular level using firefly luciferase labeled human ovarian cancer cells (ES-2-Fluc) and the bioluminescent probe PA-AL.

Take a black 96-well plate with a transparent bottom, dilute the cells to a cell suspension with a concentration of 4 × 10^5^ cells/ml, add 100 μl to each well to reach about 40,000 cells in each well, and incubate at 37°C for 12 h. Dilute the lead compound and each inhibitor to be tested to the final concentration of 200, 100, 50, 25, 12.50, 6.25, 3.13, 1.56, and 0.78 μM, and add 100 μl to each well. After mixing, transfer to a shaker at 37°C and incubate the cells with the inhibitor for 30 min. Prepare bioluminescent probe PA-AL solution with a concentration of 20 μM. Immediately after adding, put it into *in vivo* imaging for bioluminescence imaging. The bioluminescence intensity value of each hole was analyzed by the software of the living body imager, and the exposure time was 1 min. Graphpad Prism software is used to draw the IC_50_ value of each inhibitor to be tested.

### Inhibition of Vanin-1 Inhibitor on Transgenic Mice *in vivo*

In this study, the lead compound and selected superior compounds were applied to transgenic mice expressing firefly luciferase, and the inhibition of vanin-1 enzyme was investigated at the *in vivo* level using the bioluminescent probe PA-AL.

We divided FVB-luc + mice into three groups:

(1) Control group: gavage lead compound, the dose is 25 mg/kg, the gavage volume is 200 μl;
(2) Experimental group: gavage compound **a**, the dose is 50 mg/kg, the gavage volume is 200 μl;
(3) Blank group: Administer normal saline, the volume of intragastric administration is 200 μl.

After intragastric administration, mice were fed routinely for 1.5 h, and then mice in each group were anesthetized with 4% chloral hydrate solution and injected intraperitoneally with physiological saline solution of bioluminescent probe PA-AL (1 mM, 200 μl). In 55 min, the bioluminescence imaging of the whole body of each group of mice was measured every 5 min, and then the bioluminescence intensity was obtained by analyzing the images with the software of the *in vivo* imager.

### Preparation of TNBS Solution

The dose of TNBS used in this experiment is 2.0 mg/50% alcohol enema, each with a volume of 100 μl. The preparation method is 40 μl of 5% TNBS (w/v) aqueous solution, 50 μl of absolute ethanol, and 10 μl of distilled water. well mixed.

### Establishment of Animal Model

The study bred C57BL/6J mice adaptively with ordinary diet for 1 week, in an ordinary clean environment, normal light and free drinking water. The mice were randomly divided into 3 groups and numbered, fasted without water for 24 h before modeling. After weighing the mouse, use isoflurane anesthesia, insert a rubber hose with a diameter of about 0.2–0.3 cm into the mouse 3.5 cm from the anus, and slowly push 100 μl of the 2.0 mg TNBS/50% alcohol mixture solution through the hose Enter the colon, slowly pull out the plastic tube after the administration, pinch the anus with your hand, and invert it for 1 min, so that the model agent can fully penetrate the intestinal cavity of the mouse. Then put them back into the breeding cage to keep lying down, wait for the mice to wake up naturally, and create a model together. The mice in the control group were given 200 μl of lead compound solution (25 mg/kg) once a day, the mice in the experimental group were given 200 μl of compound 1c solution (50 mg/kg) once a day, and the mice in the model group were given physiological salt once a day 200 μl of aqueous solution. Eight days after modeling, the mice were sacrificed for follow-up evaluation.

### Disease Activity Index Score of Each Group of Mice

After making the model, observe and record the weight changes of each group of mice every day, and observe and record the stool characteristics and occult blood of each group of mice at the same time. DAI scores are performed on each group of mice to obtain the average value of each group of mice’s DAI score. To evaluate the disease activity of each group of mice, that is, clinical symptoms.

### Specimen Tissue Collection and Processing

After the mice were sacrificed, the intestinal cavity was cut along the edge of the mesentery, and the colon was separated 4 cm from the anus. After the steps of fixation, cutting, dehydration, paraffin embedding, sectioning, staining, and scanning imaging, the histopathological evaluation was performed.

## Result and Discussion

### HPLC Analysis

**FIGURE 1:**
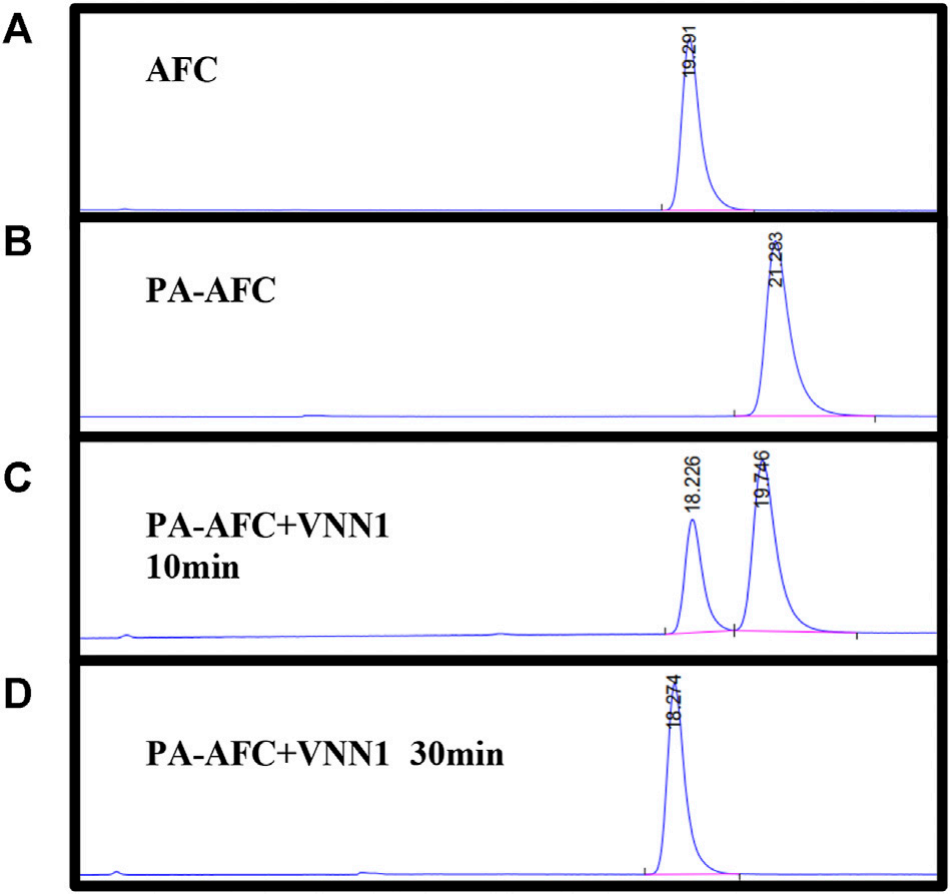
HPLC verified the reaction mechanism between fluorescent probe PA-AFC and pantothenate: **(A)** fluorophore AFC (0.2 mM); **(B)** fluorescent probe PA-AFC (0.2 mM); **(C)** probe PA-AFC (0.2 mM), 1 ml) and pantothenate (500 ng/ml, 1 ml) incubate at 37°C for 10 min, and then filter and inject; **(D)** probe PA-AFC (0.2 mM, 1 ml) and pantothenate (500 ng/ml, 1 ml) After incubating at 37°C for 30 min, filter injection.

To verify the mechanism of the off-on switch of our probe PA-AFC, an HPLC analysis was performed by a 1260 Infinity HPLC system (Agilent Technologies, Santa Clara, CA). The retention time of AFC and probe was 19.291 and 21.283 min, respectively (Figures 1A,B). When the probe was incubated with VNN1 for 10 min, the retention time showed that the fluorophore AFC peak appeared. When the probe was incubated with VNN1 for 30 min, the retention time showed that the peak of probe PA-AFC disappeared, and only the peak of the fluorophore AFC was available. As a result, probe PA-AFC can release AFC via reaction with pantetheinase (Figures 1C,D).

### Sensitivity of Fluorescent Probe PA-AFC

**FIGURE 2:**
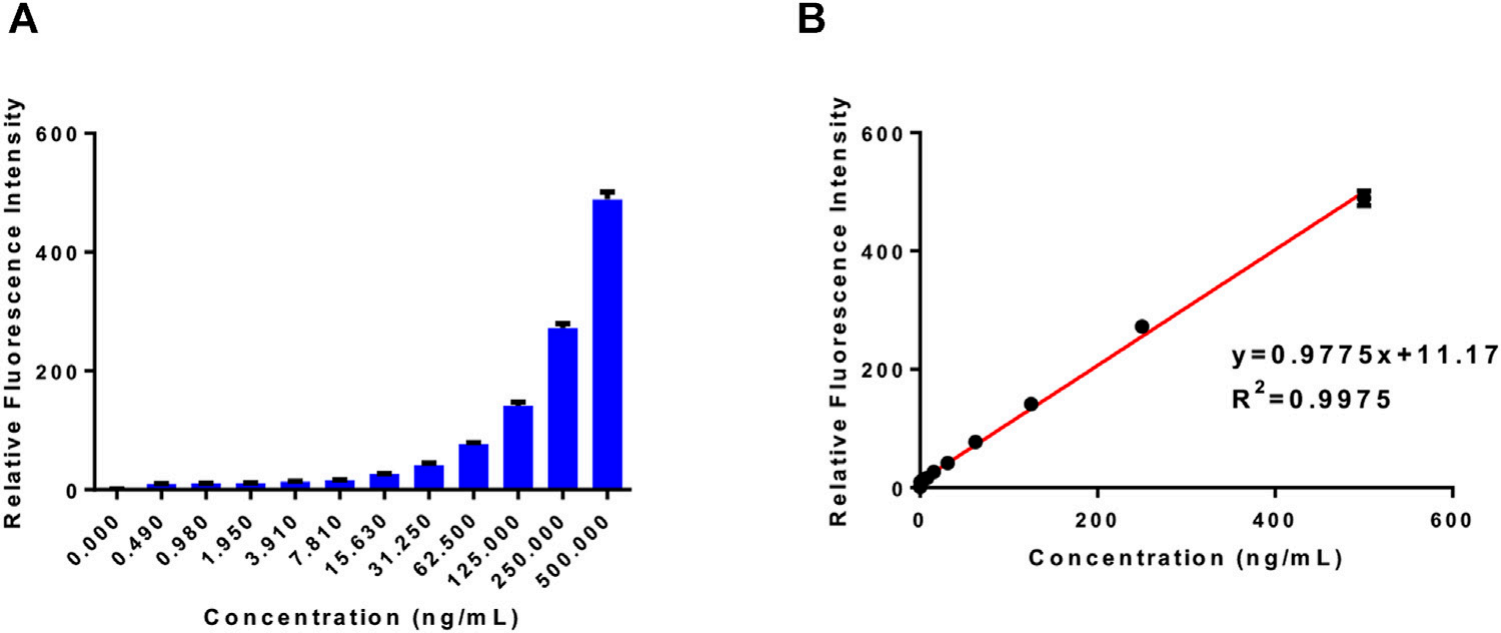
Probe sensitivity test. **(A)** The relative fluorescence intensity of the probe (20 μM) and different concentrations of human recombinant VNN1 enzyme after incubation at 37°C for 30 min; **(B)** The linear relationship between the relative fluorescence intensity and the concentration of pantothenate conforms to the linear equation *y* = 0.9775 x + 11.17, which R^2^ = 0.9975.

We incubated the 20 μM probe PA-AFC with different concentrations of human recombinant VNN1 enzyme at 37°C for 30 min, and recorded its fluorescence intensity with a microplate reader. Then, the obtained fluorescence intensity value of each concentration is divided by the intensity of the blank group to obtain the relative fluorescence intensity. In Figure 2A, we can see that the fluorescence intensity increases as the concentration of human recombinant VNN1 enzyme increases. In Figure 2B, after we linearly fitted the data in Figure A, we found that our probe has good linearity, conforming to the linear equation of *y* = 0.9775x + 11.17 and R^2^ = 0.9975. The detection limit of the probe is 1 ng/ml. This shows that we can use the probe PA-AFC to quantify the amount of VNN1, so our probe has good sensitivity.

### Selectivity of Fluorescent Probe PA-AFC

**FIGURE 3:**
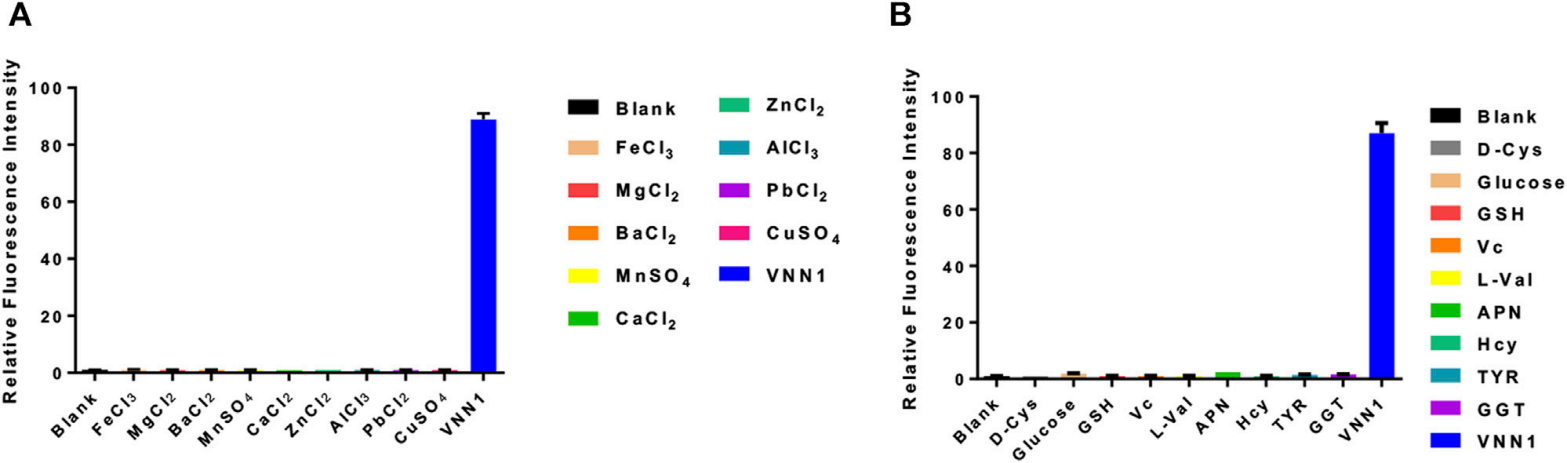
Probe *in vitro* selectivity experiment. **(A)** Relative fluorescence intensity of probe PA-AFC and various inorganic salts (1 mM), VNN1 enzyme concentration is 15 ng/ml; **(B)** Relative fluorescence intensity of probe PA-AFC and the following substance: Reduced glutathione (1 mM), D-cysteine (1 mM), glucose (1 mM), vitamin C sodium salt (1 mM), L-valine (1 mM), Homocysteine (1 mM) (1 mM), APN, (50 U/L) GGT (50 U/L), TYR (100 ng/ml), and VNN1 enzyme (15 ng/ml).

**FIGURE 4:**
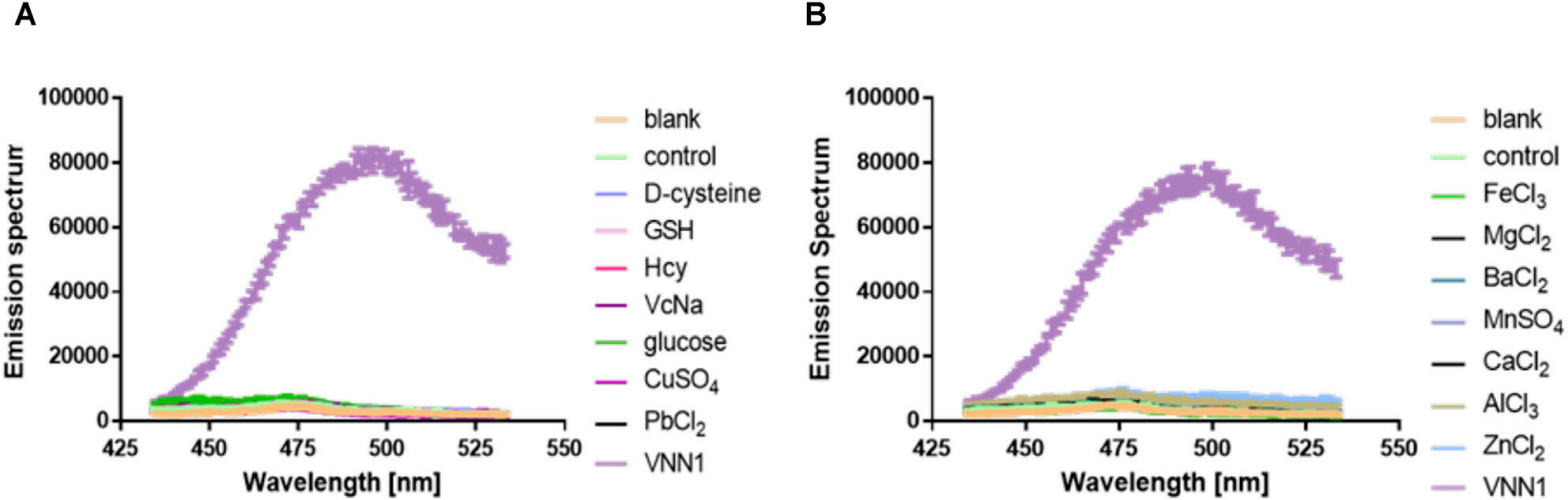
Probe *in vitro* selectivity experiment. **(A)** Emission spectrum of probe PA-AFC and the following substance: Reduced glutathione (1 mM), D-cysteine (1 mM), glucose (1 mM), vitamin C sodium salt (1 mM), Homocysteine (1 mM) (1 mM), inorganic salts (1 mM) and VNN1 enzyme (15 ng/ml); **(B)** Emission spectrum of probe PA-AFC and various inorganic salts (1 mM), VNN1 enzyme concentration is 15 ng/ml.

**FIGURE 5:**
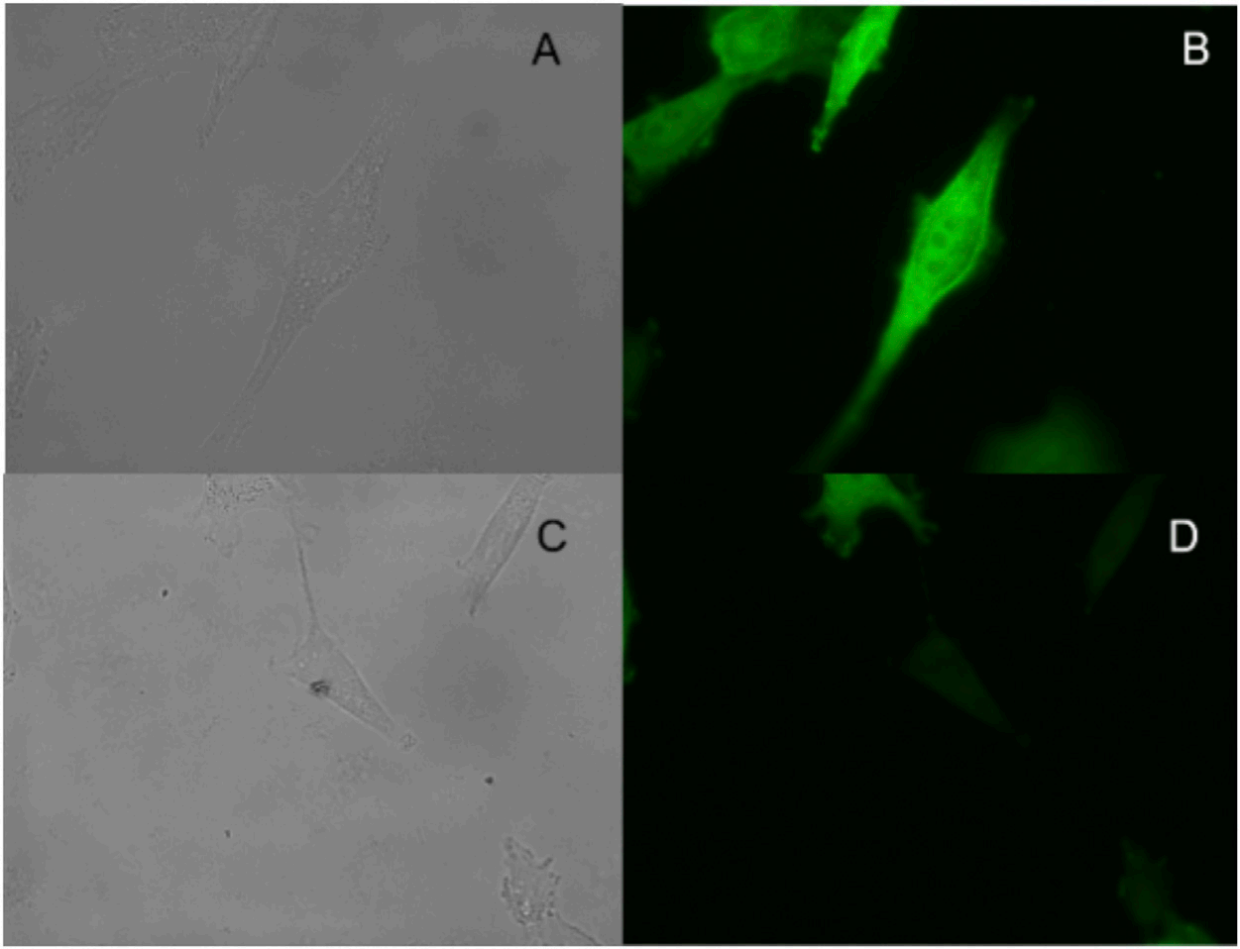
Fluorescence imaging of living ES-2-Fluc cells: top row, ES-2-Fluc cells incubated with the probe PA-AFC (20 μM) for 30 min: **(A)** bright-field, **(B)** green channel; bottom row, ES-2-Fluc cells incubated with the probe PA-AFC (20 μM) and Fe^3+^ (1 mM) for 30 min: **(C)** bright-field, **(D)** green channel.

We incubate the probe with the following substances, inorganic salt solutions, such as FeCl_3_, MgCl_2_, BaCl_2_, MnSO_4_, CaCl_2_, ZnCl_2_, AlCl_3_, PbCl_2_, CuSO_4_, and biologically active molecules such as GSH, D-cysteine, glucose, VcNa, L-valine, Hcy, and some proteases, such as APN, GGT, and TYR. Compared with 15 ng/ml VNN1 enzyme, the fluorescence intensity of other interfering substances has almost no change, which proves that our probe has good selectivity *in vitro* (Figure 3). Meanwhile, compared with 15 ng/ml VNN1 enzyme, the emission spectrum of other interfering substances has almost no change, which proves that our probe has good selectivity *in vitro* (Figure 4). As depicted in Figure 5, ES-2-Fluc cells incubated with probe PA-AFC (20 μM) for 30 min without treated with Fe^3+^ provide strong fluorescent cells. After ES-2-Fluc cells were treated with Fe^3+^ and then incubated with probe PA-AFC for 30 min, they could emit weak fluorescence imaging in the cells. These thought-provoking results confirmed again that fluorescence quenching of probe PA-AFC by Fe^3+^. The results of fluorescence quantum yield also support this conclusion (Table 2).

**TABLE 2:** Fluorescence quantum yield.

Probe	λ_ex_ (nm)	λ_em_ (nm)	Φc
PA-AFC	338	438	1.032 ± 0.0011
PA-AFC + Fe^3+^	338	438	0.049 ± 0.0013

### Inhibitory Activity *in vitro*

We used the fluorescent probe PA-AFC to evaluate the inhibitory activity of the designed and synthesized small molecule inhibitors on human recombinant VNN1 enzyme *in vitro* (Table 3). Among them, compound **a** has the best inhibitory activity, with an IC_50_ value of 20.17 μM, which is expected to become a new skeleton for vanin-1 enzyme inhibitors and drugs for the treatment of related diseases.

**TABLE 3:** Inhibitory activity of compounds *in vitro*.

Compounds	IC_50_ (μM)	Compounds	IC_50_ (μM)
Lead compound	10.36 ± 1.03 nM	**f**	46.18 ± 1.27
**a**	20.17 ± 1.32	**g**	51.48 ± 1.65
**b**	26.24 ± 0.56	**h**	84.55 ± 2.50
**c**	30.33 ± 0.11	**i**	115.47 ± 13.01
**d**	28.47 ± 0.35	**j**	138.27 ± 0.68
**e**	43.73 ± 1.07		

### Inhibitory Activity in Cellulo

We first dilute the inhibitor to three concentrations of 250, 125, and 62.5 μM, and use the MTT method and bioluminescence probe PA-AL to study the cytotoxicity of the inhibitor. As shown in Supplementary Figure S1, our inhibitor has less cytotoxicity and will not interfere with the experimental results under the experimental conditions of cell bioluminescence imaging.

Afterward, we applied the compound to ES-2-Fluc cells expressing firefly luciferase, using the bioluminescent probe PA-AL, to visually evaluate its inhibitory activity at the cellular level. Among them, compound **a** has the best inhibitory effect, with an IC_50_ value of 9.08 μM (Table 4). In addition, we found that the cellular level and enzyme level *in vitro* screening results are highly consistent in trend.

**TABLE 4:** Inhibitory activity of each compound at the cellular level.

Compounds	IC_50_ (μM)	Compounds	IC_50_ (μM)
Lead compound	3.06 ± 0.19	**f**	21.96 ± 2.66
**a**	9.08 ± 0.57	**g**	39.80 ± 0.91
**b**	12.33 ± 1.28	**h**	59.61 ± 5.27
**c**	16.47 ± 1.92	**i**	93.72 ± 8.08
**d**	15.26 ± 0.75	**j**	80.56 ± 6.21
**e**	35.76 ± 2.15		

### Inhibitory Effects of Inhibitors on Transgenic Mice *in vivo*

**FIGURE 6:**
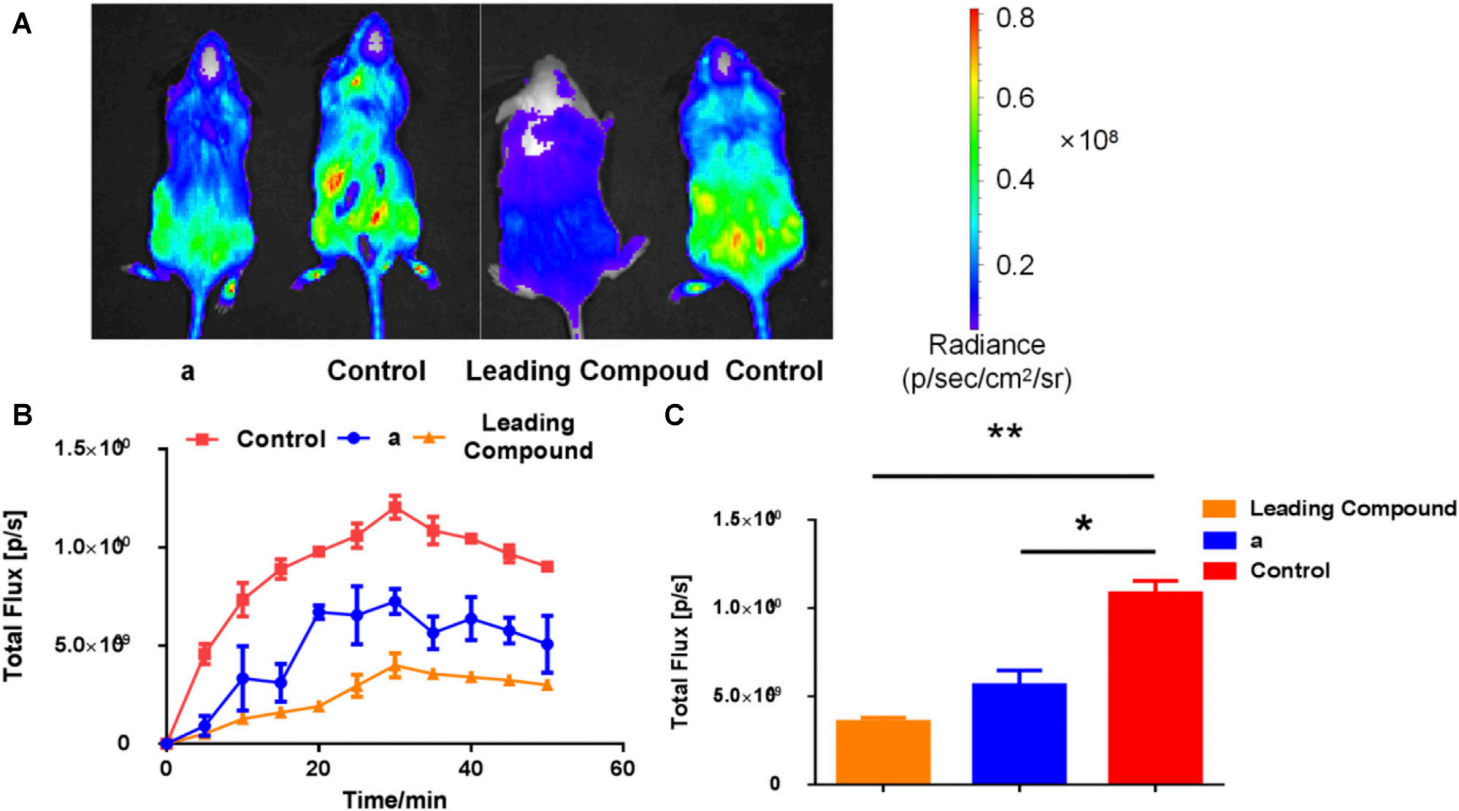
The expression of vanin-1 enzyme in FVB-luc + transgenic mice after oral administration of inhibitors, and probe (1 mM, 200 μl) was injected 1.5 h later. **(A)** Bioluminescence imaging pictures of compound **a** group (50 mg/kg) and lead compound group (25 mg/kg) 30 min after probe injection; **(B)** bioluminescence intensity in mice at different times after probe injection **(C)** Software analysis Figure 4A is the bioluminescence intensity value 30 min after the probe is injected (*n* ≥ 3; *p < 0.05, **p < 0.01 *vs*. Control).

We tested the *in vivo* activity of lead compound and the dominant compound a in transgenic mice. As shown in Figure 6, about 30 min after injection of the bioluminescence probe PA-AL, the bioluminescence intensity of each group of mice reached the peak. As shown in Figures 4A,B, at the peak, whether it is the leading compound group or the compound a group, under physiological conditions. The bioluminescence intensity of transgenic mice has been significantly reduced, which has a good inhibitory effect. As shown in Figure 4C, when it reaches the peak, the bioluminescence intensity of the lead compound group is only about one-third of that of the blank group, group 1c. The bioluminescence intensity is about one-half of the blank group.

### Application of Vanin-1 Inhibitors in Mouse Colitis Model

**FIGURE 7:**
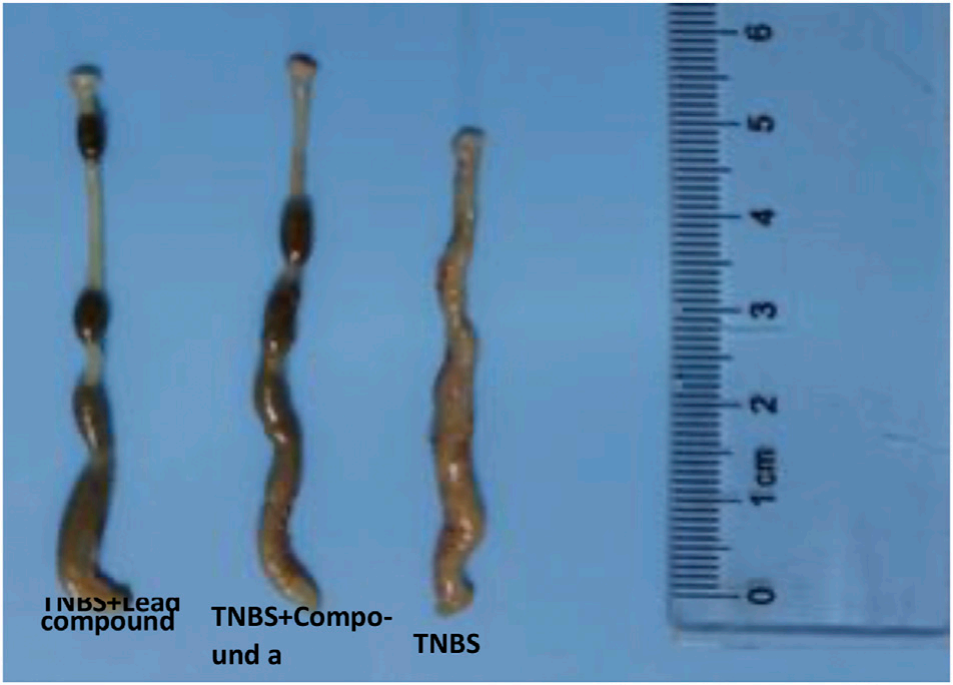
Observation of intestinal characteristics of mice in each group.

**FIGURE 8:**
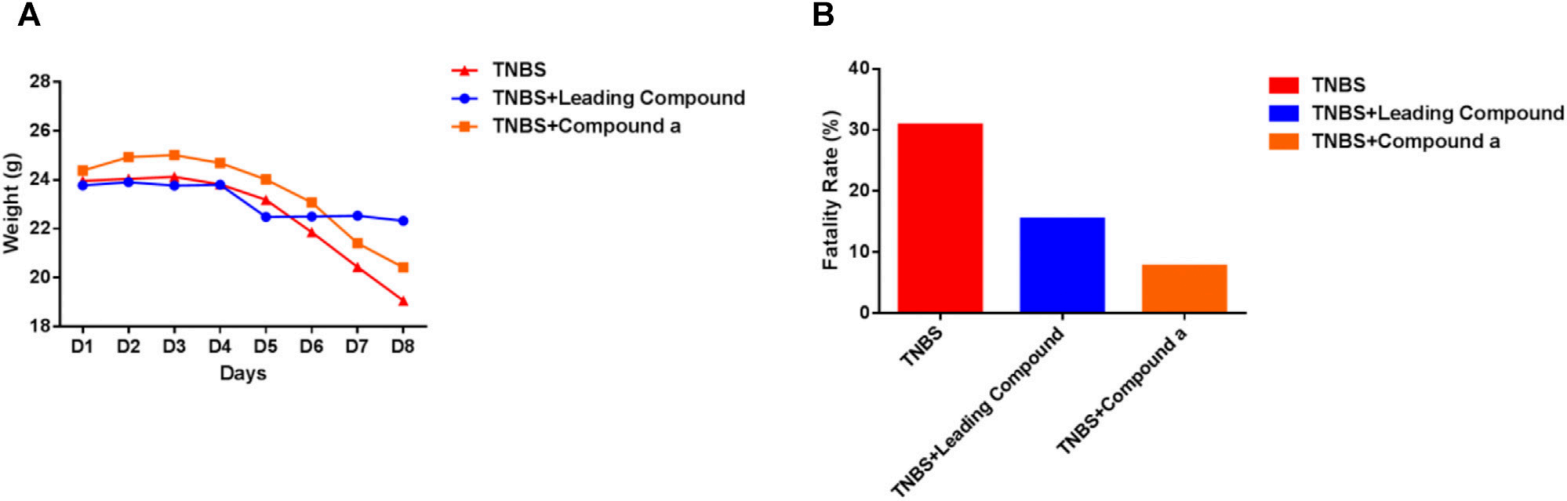
Clinical symptoms of mice in each group. **(A)** Changes in the average body weight of each group of mice during the modeling period. **(B)** Fatality rate of mice in each group (*n* = 13).

Subsequently, we explored the effects of two vanin-1/VNN1 small molecule inhibitors on mouse colitis models. We selected the chemical agent TNBS to induce colitis in mice. The subsequent exploration process was mainly carried out by observing the macroscopic clinical symptoms of each group of mice (Table 5), intestinal specimen observation (Figure 7), microscopic pathological section observation, and section inflammation score. The experimental results obtained are uniform. TNBS can indeed cause serious intestinal damage to mice, causing them to experience symptoms such as lack of food, curled up, dull coat, loose stools, bloody stools, and even death. However, this damage can be significantly improved by oral vanin-1/VNN1 small molecule inhibitors. The clinical symptoms are varied or no symptoms appear, the intestinal necrosis or atrophy is reduced, and the fatality rate is greatly reduced (Figure 8B). However, the effects of the two inhibitors are not the same (Figure 8A), which is probably caused by the difference in the inhibitory effects of the two inhibitors. The better the inhibitory effect, the more obvious the improvement of the inflammatory injury, and vice versa, the less obvious the effect.

**TABLE 5:** DAI score results of mice in each group.

Groups	Number of mice	DAI score
TNBS	9	10.0 ± 1.63
TNBS + Compound **a**	11	6.7 ± 0.95
TNBS + lead compound	12	2.8 ± 0.92

(The control group vs. the model group, the experimental group vs. the model group, the experimental group vs. the control group, ***p < 0.0001.).

**FIGURE 9:**
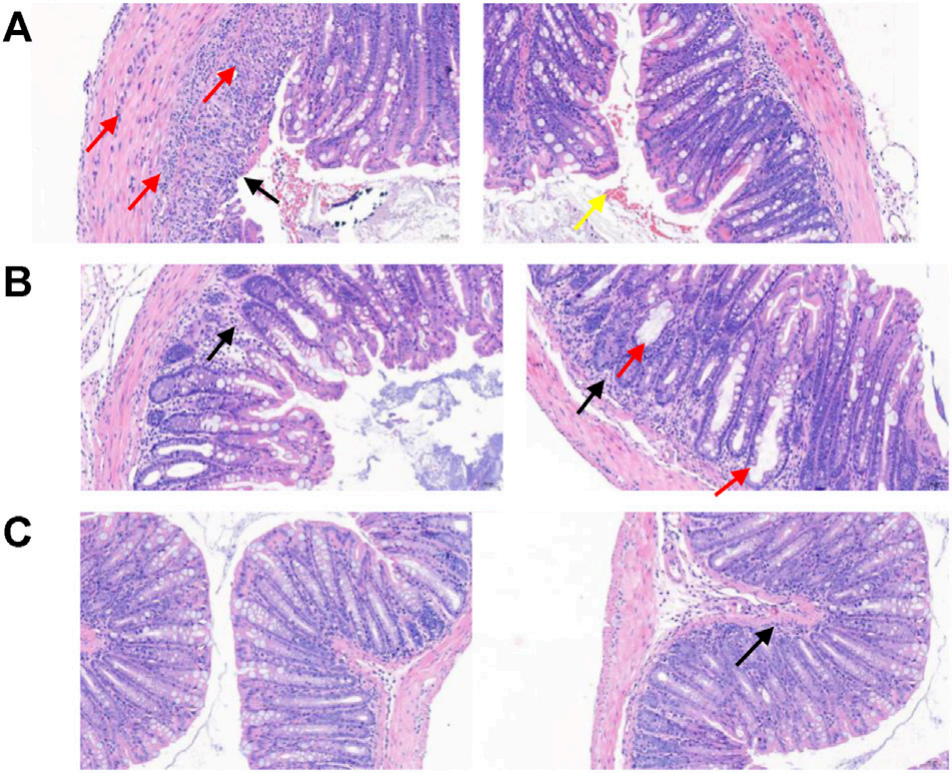
Observation of intestinal pathological sections of mice in each group. **(A)** In the model group, the intestinal pathological section of the mice was imaged. Observation and analysis showed that the tissues were local ulcers and the mucosal epithelium was missing (black arrow); the intestinal gland structure disappeared and was replaced by hyperplastic connective tissue, and a small amount of connective tissue hyperplasia was also seen in the submucosa. More inflammatory cell infiltration can be seen in the mucosal layer, submucosa, and muscle layer (red arrow), a small amount of epithelial cell necrosis, and nuclear fragmentation or pyknosis (yellow arrow). **(B)** The intestinal pathological section of the mice in the experimental group was imaged. Observation and analysis showed that the structure of each layer of the tissue was clear, the mucosal epithelium was intact, the intestinal glands were loosely arranged, and a small amount of inflammatory cell infiltration (black arrow) was seen in the lamina propria, and more intestinal glands were expanded in Irregular shape (red arrow). **(C)** The intestinal pathological section of mice in the control group was imaged. Observation and analysis showed that the structure of each layer of the tissue was clear, the mucosal epithelium was intact, the intestinal glands were abundant and arranged tightly, and a small amount of inflammatory cell infiltration (black arrow) was seen in the mucosal layer, without obvious abnormality.

After taking out the intestinal specimens of each group of mice, they were observed by naked eyes. Observation results display that in the TNBS colitis animal model, the lesions of the end of the colon are the main ones. Compared with the control group, the experimental group has obvious intestinal distortion and non-granular intestinal contents, while the model group has obvious continuous edema, transmural necrosis, and shortened intestinal atrophy (Figure 9 and Table 6).

**TABLE 6:** Intestinal inflammation scores of mice in each group.

Groups	Number of mice	DAI score
TNBS	9	10.0 ± 1.63
TNBS + Compound **a**	11	6.7 ± 0.95
TNBS + lead compound	12	2.8 ± 0.92

(The control group vs. the model group, ***p < 0.0001; the experimental group vs. the model group, **p < 0.01; the experimental group vs. the control group, *p < 0.05.).

## Conclusion

In the current study, we designed and synthesized a series of vanin-1/VNN1 small molecule inhibitors with different backbones, and used fluorescent probes and bioluminescent probes to visually evaluate their inhibitory activity at different levels, and established a set of screening inhibitors of this enzyme. In addition, we have proved that vanin-1/VNN1 does play a certain role as a pro-inflammatory agent during the occurrence of IBD. Our research results also indicate that the development of vanin-1/VNN1 inhibitors will become a new direction of effective treatment for IBD to relieve the suffering of patients.

We will continue to use visualization-based method to screen out inhibitors with enhanced inhibitory activity, and apply them to more disease models related to vanin-1/VNN1, evaluate their effects, explore their mechanisms of action, and develop diseases therapeutic drugs.

## Data Availability

The original contributions presented in the study are included in the article/Supplementary Material, further inquiries can be directed to the corresponding author.
