# Supplementary material for: Visualization-Based Discovery of Vanin-1 Inhibitors for Colitis
Source: Front Chem. 2022 Jan 28;9:809495. doi: 10.3389/fchem.2021.809495 (PMC8831383; doi:10.3389/fchem.2021.809495)
Supplement: Supplementary file 1 [file DataSheet1.docx]

Supplementary Material

Visualization-based Discovery of Vanin-1 Inhibitors for Colitis

Guankai Wang^1^, Jingjing Wang^1^, Lupei Du^1^, Minyong Li^1,*^

^1^ Department of Medicinal Chemistry, Key Laboratory of Chemical Biology (MOE), School of Pharmaceutical Sciences, Cheeloo College of Medicine, Shandong University, Jinan, Shandong 250012, China

*** Correspondence:**

Professor Minyong Li, [mli@sdu.edu.cn](mailto:mli@sdu.edu.cn)

# These authors contributed equally.

**CONTENT**

[1 General methods S3](#_Toc25740)

[2 Synthesis S3](#_Toc22158)

[3 Experiment graphs S13](#_Toc29480)

[4 NMR and MS spectra of the target compound S13](#_Toc7726)

# General methods

All chemicals and solvents were used without purification unless otherwise noted. Column chromatography was carried out on silica gel (200-300 mesh) using an eluent of ethyl acetate and cyclohexane ether. TLC analyses were conducted on silica gel plates; products were visualized using UV light (ZF-2 UV254) and I2. Mass spectral analyses were performed on an API 4000 (ESI-HRMS). NMR spectra were recorded at ^1^H (400 MHz) and ^13^C (100 MHz) on a Bruker instrument. Chemical shifts (δ values) and coupling constants (J values) are given in ppm and hertz respectively, using solvents (^1^H NMR, ^13^C NMR) as the internal standard in DMSO-*d_6_* and CDCl_3_ solution.

# Synthesis

The probe PA-AFC was prepared according to Scheme S1.

Scheme S1. Synthetic route of probe PA-AFC. Reagents and conditions:(i)D-Camphorsulfonic acid,TFA,DCM,rt,8 h; (ii) i-BuOCOCl, NMM, THF, 0˚C-rt, 12 h; (iii) H_2_O/CH_3_COOH, rt, 20 h

3-((4R)-2-(4-methoxyphenyl)-5,5-dimethyl-1,3-dioxane-4-carboxamido)propanoic acid (PA-1)

The intermediate PA-1 was synthesized by dissolving the starting materials calcium pantothenate (9.03 mmol, 2.083g) and dextro-camphorsulfonic acid (1.1 mmol, 237.6 mg) in TFA, and stirring at room temperature for 15 minutes, and then evaporating the solvent After TFA, a colorless oil was obtained. The colorless oil was dissolved in 20 mL of dichloromethane, 4-methoxybenzaldehyde dimethyl acetal (28.5 mmol, 4.33 g) was added, and the mixture was added in three portions. After 6 hours of reaction at room temperature, TLC monitored the reaction to be complete. Subsequently, 25 mL of 1M sodium carbonate solution was slowly added, and a large amount of white precipitate was formed. After the precipitate was filtered off with celite, the filtrate was washed with dichloromethane three times. Slowly add acetic acid to the water layer to adjust the pH to 4-5. Then it was extracted three times with dichloromethane, the organic phase was retained, and dried over anhydrous sodium sulfate for 2 h. The anhydrous sodium sulfate was filtered off, the filtrate was collected, and the solvent was evaporated to obtain 1.21 g of a white flake solid, with a yield of 28.3%. 1H NMR (400 MHz, DMSO-*d_6_*) δ 11.95 (s, 1H), 7.47 (d, J = 5.9 Hz, 1H), 7.46–7.36 (m, 2H), 6.93 (d, J = 8.7 Hz, 2H) , 5.53 (s, 1H), 4.09 (s, 1H), 3.76 (s, 3H), 3.69–3.60 (m, 2H), 3.30–3.20 (m, 2H), 2.47–2.33 (m, 2H), 1.01 (t, J = 23.6 Hz, 6H). ESI-MS: m/z[2M+Na]+ cacd for C34H46N2O12Na+ 697.29, found 697.19; m/z[M+Na]+ cacd for C17H23NO6Na+ 360.1 found 360.55. Melting point: 155.3-157.1 ^o^C.

2-(4-methoxyphenyl)-5,5-dimethyl-N-(3-oxo-3-((2-oxo-4-(trifluoromethyl)-2H-chromen-7-yl)amino)propyl)-1,3-dioxane-4-carboxamide (PA-2)

The synthesis of intermediate PA-2 is to dissolve intermediate PA-1 (200 mg, 0.59 mmol) in 2 mL of dry tetrahydrofuran. Add N-methylmorpholine (67 μL, 0.59 mmol) to the solution and place in an ice bath. To the solution, i-BuOCOCl (75 μL, 0.59 mmol) was slowly added dropwise, and the mixture was stirred at 0 ^o^C for 0.5 h. Then 7-amino-4-trifluoromethylcoumarin (45 mg, 0.20 mmol) was dissolved in 2 mL of tetrahydrofuran and added dropwise to the reaction solution. The mixture was stirred overnight at room temperature. Then the solvent was evaporated. The mixture was diluted with ethyl acetate and washed with saturated sodium bicarbonate solution, water and brine (50 mL), and dried over anhydrous sodium sulfate for 2 h. Filter off anhydrous sodium sulfate, collect the filtrate, and evaporate the solvent. The crude product was separated and purified. 59 mg of pale-yellow solid was obtained, yield = 54.6%. 1H NMR (400 MHz, DMSO-*d_6_*) δ 11.95 (s, 1H), 7.47 (d, J = 5.9 Hz, 1H), 7.46–7.36 (m, 2H), 6.93 (d, J = 8.7 Hz, 2H) , 5.53 (s, 1H), 4.09 (s, 1H), 3.76 (s, 3H), 3.69–3.60 (m, 2H), 3.30–3.20 (m, 2H), 2.47–2.33 (m, 2H), 1.01 (t, J = 23.6 Hz, 6H). ESI-MS: m/z[M-H]-cacd for C27H26F3N2O7-547.17, found 547.39. Melting point: 120.6-121.4 ^o^C.

2,4-dihydroxy-3,3-dimethyl-N-(3-oxo-3-((2-oxo-4-(trifluoromethyl)-2H-chromen-7-yl)amino)propyl)butanamide (PA-AFC)

The target compound PA-AFC is synthesized by deprotecting the intermediate PA-2. Dissolve PA-2 (50 mg, 91.16 μmol) with 80% acetic acid solution, stir overnight at room temperature, spin off the solvent, and separate and purify the crude product, 21 mg of pale-yellow solid was obtained with a yield of 53.5%. 1H NMR (400 MHz, DMSO-*d_6_*) δ 10.55 (s, 1H), 7.88 (t, J = 8.3 Hz, 1H), 7.79 (t, J = 5.8 Hz, 1H), 7.73–7.61 (m, 1H) , 7.52 (dd, J = 8.9, 2.0 Hz, 1H), 6.89 (s, 1H), 5.36 (t, J = 12.3 Hz, 1H), 4.46 (t, J = 5.5 Hz, 1H), 3.72 (d, J = 5.5 Hz, 1H), 3.44 (ddd, J = 23.1, 14.8, 6.5 Hz, 2H), 3.29 (dd, J = 10.5, 5.8 Hz, 1H), 3.18 (dd, J = 10.4, 5.3 Hz, 1H ), 2.67–2.57 (m, 2H), 0.81 (dd, J = 17.4, 6.3 Hz, 6H). 13C NMR (101 MHz, DMSO-*d_6_*) δ 173.50, 171.20, 159.11, 155.19, 143.82, 139.51, 125.87, 116.42, 114.80, 114.75, 108.58, 106.59, 75.47, 68.47, 36.86, 34.87, 21.40, 20.70. ESI-HRMS: m/z [M + H]+cacd for C19H22F3N2O6+431.1430, found 431.1422. HPLC analysis: Retention time = 11.332 min, C8 reversed-phase column (250 mm × 4.6 mm, 5 μm, Phenomenex), H2O (containing 0.1% triethylamine) as solvent A and CH3OH as solvent B, the gradient program was as follows: 50% A/50% B, 1 mL/min, purity 99.2%. Melting point: 92.4-93.6 ^o^C.

The inhibitors **a-h** were prepared according to Scheme S2

Scheme S2. Synthetic route of inhibitors a-h. Reagents and conditions: (i) p-Toluenesulfonic acid, anhydrous methanol, rt, 3 h; (ii) Sodium cyanoborohydride or sodium borohydride, anhydrous methanol, rt, 6 h; (iii) NaOH, H2O/MeOH, HCl, rt, 12 h; (iv) HATU, TEA, DMF, 60 ˚C, 16 h.

ethyl 4-((pyridin-3-ylmethyl)amino)benzoate (1)

The synthesis of intermediate 1 is to combine the starting materials 3-pyridinecarboxaldehyde (71.3 mg, 665.89 μmol), ethyl 4-aminobenzoate (100 mg, 605.36 μmol) and a catalytic amount of p-toluenesulfonic acid (2.08 mg, 12.11 μmol). Dissolve in 8 mL of anhydrous methanol, stir for 3 h at room temperature, divide sodium cyanoborohydride (133.2 mg, 2.12 mmol) into the stirred reaction solution at intervals of 1 h each time, and react 3. h. The organic solvent was evaporated from the reaction solution, 10 mL of distilled water was added, and the mixture was extracted three times with dichloromethane. The organic phases were combined and washed three times with distilled water and three times with saturated sodium chloride solution. The washed organic phase was dried with anhydrous sodium sulfate for 2 h. The anhydrous sodium sulfate solid was filtered off, and the organic solvent was evaporated. The crude product was separated and purified to obtain 56.5 mg of a white oily substance with a yield of 36.4%. 1H NMR (400 MHz, DMSO-*d_6_*) δ 8.58 (s, 1H), 8.46 (d, J = 4.6 Hz, 1H), 7.70 (dd, J = 23.2, 7.8 Hz, 3H), 7.42 – 7.29 (m, 1H), 7.12 (t, J = 5.7 Hz, 1H), 6.64 (d, J = 7.9 Hz, 2H), 4.38 (d, J = 5.8 Hz, 2H), 4.19 (q, J = 6.9 Hz, 2H) , 1.26 (t, J = 7.0 Hz, 3H). ESI-MS: m/z [M + H]+cacd for C15H17N2O2+257.1, found 257.2.

4-((pyridin-3-ylmethyl)amino)benzoic acid (2)

The synthesis of intermediate 2 is to dissolve intermediate 1 (50 mg, 195.08 μmol) in 5 mL methanol, weigh out solid sodium hydroxide (23.41 mg, 585.24 μmol), dissolve it with 5 mL of distilled water, and add dropwise to the methanol solution of 1. The reaction was stirred at room temperature for 12 h. The methanol in the reaction solution was distilled off, leaving the water phase, which was washed three times with dichloromethane, and the water phase was retained. Adjust the pH value of the reaction solution with 1M hydrochloric acid solution until there is solid precipitation (pH=about 4). The reaction solution was filtered, the filter cake was retained and washed with acetone, and dried in a vacuum pump to obtain 31.25 mg of a white powdery solid with a yield of 70.2%. Melting point: 223.9-224.3 ^o^C. 1H NMR (400 MHz, DMSO-*d_6_*) δ 12.03 (s, 1H), 8.58 (s, 1H), 8.42 (t, J = 21.2 Hz, 1H), 7.69 (dd, J = 32.2, 7.7 Hz, 3H) , 7.41–7.28 (m, 1H), 7.05 (t, J = 5.6 Hz, 1H), 6.61 (d, J = 7.9 Hz, 2H), 4.38 (d, J = 5.7 Hz, 2H). ESI-MS: m/z [M-H]-cacd for C13H11N2O2- 227.1, found 227.3.

(4-((pyridin-3-ylmethyl)amino)phenyl)(8-oxa-2-azaspiro[4.5]decan-2-yl)methanone (a)

The target compound a was synthesized by dissolving intermediate 2 (50 mg, 219.06 μmol) and HATU (99.95 mg, 262.87 μmol) in 10 mL DMF, and reacting for 2 hours under nitrogen protection and 60℃ oil bath. Then under the protection of nitrogen, add 8-oxa-2-azaspiro[4.5]decane (37.12 mg, 262.87 μmol), then add triethylamine (106.28 μL, 766.70 μmol), and stir for 14 h in an oil bath at 60℃ . After the reaction, the organic solvent was distilled off, 30 mL of distilled water was added, and dichloromethane extracted three times, and the organic phases were combined. The organic phase was washed three times with distilled water, and after three times with saturated sodium chloride solution, dried with anhydrous sodium sulfate for 2 h. The anhydrous sodium sulfate solid was filtered off, the filtrate was collected, and the organic solvent was evaporated. The crude product was separated and purified to obtain a pale yellow solid 26.88 mg, with a yield of 34.9%. Melting point: 113.2-114.7 ^o^C. 1H NMR (400 MHz, DMSO-*d_6_*) δ 8.82–8.22 (m, 2H), 7.75 (d, J = 7.6 Hz, 1H), 7.56–7.13 (m, 3H), 6.74 (s, 1H), 6.58 ( d, J = 8.3 Hz, 2H), 4.34 (d, J = 5.8 Hz, 2H), 3.44 (d, J = 55.6 Hz, 6H), 1.39 (dt, J = 119.0, 87.3 Hz, 8H). 13C NMR (101 MHz, DMSO-*d_6_*) δ 169.23, 150.31, 149.34, 148.56, 135.69, 135.54, 129.66, 124.08, 123.97, 111.57, 100.00, 64.72, 44.16, 40.61, 40.40, 40.19, 39.99, 39.78, 39.57, 39.31 , 34.92, 29.48, 14.42 .ESI-HRMS: m/z [M+H]+ cacd for C21H26N3O2+352.2025, found 352.2029. HPLC analysis :Retention time = 11.332 min, C8 reversed-phase column (250 mm × 4.6 mm, 5 μm, Phenomenex), H2O (containing 0.1% triethylamine) as solvent A and CH3OH as solvent B, the gradient program was as follows: 50% A/50% B, 1 mL/min, purity 99.7%.

(4-((pyridin-3-ylmethyl)amino)phenyl)(7-oxa-2-azaspiro[3.5]nonan-2-yl)methanone (b)

The synthesis of the target compound b is based on the intermediate 2 (50 mg, 219.06 μmol) and 7-oxa-2-azaspiro[3.5]nonane (33.43 mg, 262.87 μmol) as raw materials, and the synthesis is carried out according to the method of 1c. 27.93 mg of yellowish solid was obtained, and the yield was 37.9%. Melting point: 126.1-126.5 ^o^C. 1H NMR (400 MHz, DMSO-*d_6_*) δ 8.53 (d, J = 50.5 Hz, 2H), 7.75 (s, 1H), 7.41 (d, J = 25.5 Hz, 3H), 6.88 (s, 1H), 6.61 (s, 2H), 4.37 (s, 2H), 4.04 (s, 2H), 3.73 (s, 2H), 3.51 (s, 4H), 1.68 (s, 4H).13C NMR (101 MHz, DMSO-*d_6_*) δ 169.59, 151.06, 149.34, 148.60, 135.58, 135.50, 130.04, 123.99, 120.68, 111.71, 64.40, 63.57, 59.13, 44.05, 40.65, 40.44, 40.23, 40.02, 39.81, 39.60, 39.39, 36.07- ESI, HRMS: m/z [M+H]+cacd for C20H24N3O2+ 338.1869, found 338.1865. HPLC analysis: Retention time = 14.284 min, C8 reversed-phasecolumn (250 mm×4.6 mm, 5 μm, Phenomenex), H2O (containing 0.1 % triethylamine) as solvent A and CH3OH as solvent B, the gradient program was as follows: 55% A/45% B, 1 mL/min, purity 99.8%.

ethyl 4-((pyrazin-2-ylmethyl)amino)benzoate (3)

The synthesis of intermediate 3 is to synthesize the starting materials pyrazine-2-carbaldehyde (71.98 mg, 665.89 μmol) and ethyl 4-aminobenzoate (100 mg, 605.36 μmol) according to the method of intermediate 1. 67.56 mg of yellow powdery solid was obtained, and the yield was 43.3%. Melting point: 115.2-116.0 ^o^C. 1H NMR (400 MHz, DMSO-*d_6_*) δ 8.83–8.35 (m, 3H), 7.69 (d, J = 8.2 Hz, 2H), 7.21 (t, J = 5.7 Hz, 1H), 6.67 (d, J = 8.2 Hz, 2H), 4.53 (d, J = 6.0 Hz, 2H), 4.20 (q, J = 7.0 Hz, 2H), 1.26 (t, J = 7.1 Hz, 3H).ESI-MS: m/z [M+H]+cacd for C14H16N3O2+258.1, found 258.1.

4-((pyrazin-2-ylmethyl)amino)benzoic acid (4)

The synthesis of intermediate 4 was based on intermediate 3 (50 mg, 194.33 μmol) as the raw material and was synthesized according to the method of intermediate 2 to obtain 30.78 mg of pale-yellow powder with a yield of 69.1%. Melting point: 286.8-287.3 ^o^C. 1H NMR (400 MHz, DMSO-*d_6_*) δ 8.70–8.55 (m, 2H), 8.50 (t, J = 11.0 Hz, 1H), 7.63 (t, J = 12.1 Hz, 2H), 6.60 (t, J = 6.2 Hz, 1H), 6.48 (dd, J = 19.0, 8.6 Hz, 2H), 4.45 (d, J = 6.2 Hz, 2H). ESI-MS: m/z [M-H]- cacd for C12H10N3O2- 228.1 , found 228.3.

(4-((pyrazin-2-ylmethyl)amino)phenyl)(8-oxa-2-azaspiro[4.5]decan-2-yl)methanone (c)

The synthesis of target compound c is based on intermediate 4 (50 mg, 218.11 μmol) and 8-oxa-2-azaspiro[4.5]decane (36.96 mg, 261.74 μmol) as raw materials, and proceeded according to the method of target compound a synthesis. 24.67 mg of orange solid was obtained, and the yield was 32.1%. Melting point: 173.4-173.8 °C. 1H NMR (400 MHz, DMSO-*d_6_*) δ 8.63 (dd, J = 37.5, 25.8 Hz, 3H), 7.33 (d, J = 8.0 Hz, 2H), 6.83 (s, 1H), 6.61 (d, J = 8.1 Hz, 2H), 4.49 (d, J = 5.8 Hz, 2H), 3.52 (s, 6H), 1.82–1.23 (m, 8H).13C NMR (101 MHz, DMSO-*d_6_*) δ 169.11, 155.41, 150.15 , 144.51, 144.01, 143.72, 129.68, 124.30, 111.58, 64.69, 59.88, 56.64, 46.62, 44.69, 35.27, 34.61. ESI-HRMS: m/z [M+H]+ cacd for C20H25N4O2+ 353.1978, found 353.1976. HPLC analysis :Retention time = 11.118 min, C8 reversed-phase column (250 mm × 4.6 mm, 5 μm, Phenomenex), H2O (containing 0.1% triethylamine) as solvent A and CH3OH as solvent B, the gradient program was as follows: 55% A/45% B, 1 mL/min, purity 99.6%.

(4-((pyrazin-2-ylmethyl)amino)phenyl)(7-oxa-2-azaspiro[3.5]nonan-2-yl)methanone (d)

The target compound d was synthesized using intermediate 4 (50 mg, 218.11 μmol) and 7-oxa-2-azaspiro[3.5]nonane (33.29 mg, 261.74 μmol) as raw materials, and synthesized according to the method of target compound a. 25.73 mg of orange solid was obtained, and the yield was 34.9%. Melting point: 191.6-191.9 ^o^C. 1H NMR (400 MHz, DMSO-*d_6_*) δ 8.63 (dd, J = 34.7, 28.9 Hz, 3H), 7.44 (d, J = 7.8 Hz, 2H), 6.96 (s, 1H), 6.62 (d, J = 7.8 Hz, 2H), 4.50 (d, J = 5.1 Hz, 2H), 4.03 (s, 2H), 3.72 (s, 2H), 3.51 (s, 4H), 1.68 (s, 4H). 13C NMR (101 MHz, DMSO-*d_6_*) δ 169.53, 155.29, 150.92, 144.53, 143.97, 143.74, 130.06, 129.10, 120.87, 120.34, 113.02, 111.72, 64.38, 63.20, 59.10, 46.49, 36.03, 35.95, 35.31, 33.26.ESI-HRMS : m/z [M+H]+ cacd for C19H23N4O2+ 339.1821, found 339.1818. HPLC analysis :Retention time = 9.190 min, C8 reversed-phase column (250 mm × 4.6 mm, 5 μm, Phenomenex), H2O (containing 0.1 % triethylamine) as solvent A and CH3OH as solvent B, the gradient program was as follows: 55% A/45% B, 1mL/min, purity 99.3%.

methyl 5-((pyridin-3-ylmethyl)amino)picolinate (5)

The synthesis of intermediate 5 is to combine the starting materials 3-pyridinecarboxaldehyde (77.44 mg, 722.96 μmol), methyl 5-aminopicolinate (100 mg, 657.23 μmol) and a catalytic amount of p-toluenesulfonic acid (2.26 mg, 13.14 μmol) were dissolved in 8 mL of anhydrous methanol, stir for 4 h at room temperature, add sodium borohydride (87.03 mg, 2.30 mmol) to the stirred reaction solution in portions, and add another time after the bubbles disappear each time. Reaction for 5 h after all addition. The organic solvent was evaporated from the reaction solution, 10 mL of distilled water was added, and the mixture was extracted three times with dichloromethane. The organic phases were combined and washed three times with distilled water and three times with saturated sodium chloride solution. The washed organic phase was dried with anhydrous sodium sulfate for 2 h. The anhydrous sodium sulfate solid was filtered off, and the organic solvent was evaporated. The crude product was separated and purified to obtain 60.34 mg of colorless oil with a yield of 37.7%. 1H NMR (400 MHz, DMSO-*d_6_*) δ 8.60 (s, 1H), 8.48 (d, J = 4.6 Hz, 1H), 8.09 (s, 1H), 7.78 (dd, J = 13.3, 8.2 Hz, 2H) , 7.38 (t, J = 6.1 Hz, 2H), 6.97 (d, J = 8.8 Hz, 1H), 4.43 (d, J = 5.9 Hz, 2H), 3.77 (s, 3H).ESI-MS: m/ z [M + H]+ cacd for C13H14N3O2+244.1, found 244.1.

5-((pyridin-3-ylmethyl)amino)picolinic acid (6)

The synthesis of intermediate 6 is to dissolve intermediate 5 (50 mg, 205.54 μmol) in 5 mL methanol, weigh out the solid sodium hydroxide (24.66 mg, 616.61 μmol), dissolve it with 5 mL distilled water, and add dropwise to the methanol solution of 5. The reaction was stirred at room temperature for 10 h. The methanol in the reaction solution was distilled off, leaving the water phase, which was washed three times with dichloromethane, and the water phase was retained. Adjust the pH value of the reaction solution to neutral or slightly acidic (pH=about 7) with 1M hydrochloric acid solution. The water in the solution was distilled off and dried in a vacuum pump to obtain 49.98 mg of a pale yellow powdery solid with a yield of 96.8%. Melting point: 165.5-165.9 ^o^C. 1H NMR (400 MHz, DMSO-*d_6_*) δ 8.55 (d, J = 29.4 Hz, 1H), 8.45 (d, J = 4.2 Hz, 1H), 7.89 (d, J = 19.8 Hz, 1H), 7.74 (t , J = 7.4 Hz, 2H), 7.39–7.30 (m, 1H), 6.92 (d, J = 8.3 Hz, 2H), 4.37 (d, J = 5.2 Hz, 2H).ESI-MS: m/z [ M+H]+ cacd for C12H12N3O2+ 230.1, found 230.3.

(5-((pyridin-3-ylmethyl)amino)pyridin-2-yl)(8-oxa-2-azaspiro[4.5]decan-2-yl)methanone (e)

The synthesis of the target compound e is based on the intermediate 6 (50 mg, 199.03 μmol) and 8-oxa-2-azaspiro[4.5]decane (33.73 mg, 238.83 μmol) as raw materials, and proceeded according to the synthesis method of the target compound a. 22.78 mg of pale yellow powdered solid was obtained, and the yield was 32.5%. Melting point: 176.5-177.2 ^o^C. 1H NMR (400 MHz, DMSO-*d_6_*) δ 8.61 (s, 1H), 8.48 (s, 1H), 7.98 (d, J = 7.0 Hz, 1H), 7.78 (d, J = 7.3 Hz, 1H), 7.59 (d, J = 8.1 Hz, 1H), 7.43–7.33 (m, 1H), 7.18–6.81 (m, 2H), 4.40 (d, J = 4.7 Hz, 2H), 3.83 (t, J = 7.0 Hz, 1H), 3.63–3.46 (m, 5H), 1.77 (dd, J = 13.7, 6.8 Hz, 2H), 1.58–1.38 (m, 4H), 1.24 (s, 2H).13C NMR (101 MHz, DMSO-*d_6_*) δ 164.45, 149.18, 148.77, 141.97, 139.88, 135.26, 134.59, 125.30, 125.18, 124.07, 118.27, 64.80, 64.70, 43.47, 40.63, 40.42, 40.21, 40.00, 39.79, 39, 34.85, 39.37 , 29.48.ESI-HRMS: m/z [M+H]+ cacd for C20H25N4O2+ 353.1978, found 353.1969. HPLC analysis :Retention time = 8.840 min, C8 reversed-phase column (250 mm × 4.6 mm, 5 μm, Phenomenex ), H2O (containing 0.1% triethylamine) as solvent A and CH3OH as solvent B, the gradient program was as follows: 55% A/45% B, 1mL/min, purity 97.2%.

(5-((pyridin-3-ylmethyl)amino)pyridin-2-yl)(7-oxa-2-azaspiro[3.5]nonan-2-yl)methanone (f)

The target compound f was synthesized using intermediate 6 (50 mg, 218.11 μmol) and 7-oxa-2-azaspiro[3.5]nonane (33.29 mg, 261.74 μmol) as raw materials, and synthesized according to the method of target compound a. 25.73 mg of yellow powdery solid was obtained, and the yield was 34.9%. Melting point: 202.0-202.3 ^o^C. 1H NMR (400 MHz, DMSO-*d_6_*) δ 8.53 (d, J = 49.6 Hz, 2H), 7.93 (d, J = 29.5 Hz, 1H), 7.73 (dd, J = 19.9, 7.9 Hz, 2H), 7.37 (s, 1H), 7.18 –6.93 (m, 2H), 4.40 (d, J = 4.1 Hz, 2H), 4.26 (s, 2H), 3.72 (s, 2H), 3.51 (s, 4H), 1.68 ( s, 4H).13C NMR (101 MHz, DMSO-*d_6_*) δ 165.45, 149.40, 148.76, 146.14, 139.98, 135.60, 134.96, 133.85, 124.82, 124.04, 117.89, 64.92, 64.47, 58.94, 43.71, 36.05, 58.94, 43.71, 36.05 ESI-HRMS: m/z [M+H]+ cacd for C19H23N4O2+ 339.1821, found 339.1817. HPLC analysis : Retention time = 12.117 min, C8 reversed-phase column (250 mm × 4.6 mm, 5 μm, Phenomenex), H2O (Containing 0.1% triethylamine) as solvent A and CH3OH as solvent B, the gradient program was as follows: 55% A/45% B, 1mL/min, purity 98.52%.

methyl 5-((pyrazin-2-ylmethyl)amino)picolinate (7)

The synthesis of intermediate 7 is to synthesize the starting materials pyrazine-2-carbaldehyde (78.15 mg, 722.96 μmol) and 5-aminopicolinate (100 mg, 657.23 μmol) according to the method of intermediate 3a. 65.38 mg of brown-yellow powdery solid was obtained, and the yield was 40.7%. Melting point: 135.7-136.4 ^o^C. 1H NMR (400 MHz, DMSO-*d_6_*) δ 8.80-8.43 (m, 3H), 8.12 (s, 1H), 7.79 (t, J = 9.0 Hz, 1H), 7.45 (t, J = 5.6 Hz, 1H) , 7.00 (d, J = 8.5 Hz, 1H), 4.58 (d, J = 5.9 Hz, 2H), 3.77 (s, 3H). ESI-MS: m/z [M + H]+cacd for C12H13N4O2+ 245.1 , found 245.3.

5-((pyrazin-2-ylmethyl)amino)picolinic acid (8)

The synthesis of intermediate 8 is based on intermediate 4a (50 mg, 204.70 μmol) as the raw material, and the synthesis is carried out according to the method of intermediate 3b to obtain 49.54 mg of a red-brown powdery solid with a yield of 96.0%. Melting point: 296.3-296.7 ^o^C. 1H NMR (400 MHz, DMSO-*d_6_*) δ 8.82–8.42 (m, 3H), 8.05 (s, 1H), 7.76 (d, J = 8.4 Hz, 1H), 7.37 (s, 1H), 6.99 (d, J = 8.4 Hz, 1H), 4.55 (d, J = 5.4 Hz, 2H). ESI-MS: m/z [M+H]+ cacd for C11H11N4O2+ 231.1, found 231.2.

(5-((pyrazin-2-ylmethyl)amino)pyridin-2-yl)(8-oxa-2-azaspiro[4.5]decan-2-yl)methanone (g)

The synthesis of the target compound g is based on the intermediate 8 (50 mg, 198.25 μmol) and 8-oxa-2-azaspiro[4.5]decane (33.59 mg, 237.90 μmol) as raw materials, and proceeded according to the synthesis method of the target compound a. 23.18 mg of yellow powdered solid was obtained, and the yield was 33.1%. Melting point: 123.6-124.1 ^o^C. 1H NMR (400 MHz, DMSO-*d_6_*) δ 8.84–8.42 (m, 3H), 8.01 (d, J = 9.2 Hz, 1H), 7.58 (d, J = 8.6 Hz, 1H), 7.10 (t, J = 5.9 Hz, 1H), 7.02 (d, J = 6.6 Hz, 1H), 4.54 (d, J = 5.6 Hz, 2H), 3.83 (t, J = 7.0 Hz, 1H), 3.61 – 3.49 (m, 5H) , 1.77 (dd, J = 14.0, 6.8 Hz, 2H), 1.58-1.40 (m, 5H), 1.24 (s, 1H).13C NMR (101 MHz, DMSO-*d_6_*) δ 166.01, 154.74, 145.69, 144.61, 144.12, 143.91, 141.84, 133.40, 133.32, 125.46, 118.02, 64.70, 59.68, 57.10, 47.48, 46.06, 45.38, 37.73, 35.29, 34.88. ESI-HRMS: m/z [M + H]+cacd for C19H24N5O2+ 354.1930 , found 354.1931. HPLC analysis :Retention time = 15.094 min, C8 reversed-phase column (250 mm × 4.6 mm, 5 μm, Phenomenex), H2O (containing 0.1% triethylamine) as solvent A and CH3OH as solvent B, the gradient program was as follows: 55% A/45% B, 1mL/min, purity 99.52%.

(5-((pyrazin-2-ylmethyl)amino)pyridin-2-yl)(7-oxa-2-azaspiro[3.5]nonan-2-yl)methanone (h)

The synthesis of the target compound h is based on the intermediate 8 (50 mg, 198,25 μmol) and 7-oxa-2-azaspiro[3.5]nonane (30.26 mg, 237.90 μmol) as raw materials, according to the synthesis method of target compound a. 22.47 mg of pale yellow powdery solid was obtained, and the yield was 33.4%. Melting point: 104.6-105.0 ^o^C. 1H NMR (400 MHz, DMSO-*d_6_*) δ 8.77 – 8.41 (m, 3H), 8.00 (s, 1H), 7.71 (d, J = 8.4 Hz, 1H), 7.19 (s, 1H), 7.02 (d, J = 8.7 Hz, 1H), 4.54 (d, J = 5.4 Hz, 2H), 4.27 (s, 2H), 3.72 (s, 2H), 3.51 (d, J = 4.3 Hz, 4H), 1.73 (d, J = 35.5 Hz, 4H).13C NMR (101 MHz, DMSO-*d_6_*) δ 165.43, 154.64, 146.13, 144.62, 144.10, 143.93, 140.14, 133.90, 124.81, 117.91, 64.92, 64.47, 58.94, 45.98, 36.05, 33.52 .ESI-HRMS: m/z [M + H]+ cacd for C18H22N5O2+ 340.1773, found 340.1769. HPLC analysis :Retention time = 8.137 min, C8 reversed-phase column (250 mm × 4.6 mm, 5 μm, Phenomenex), H2O (containing 0.1% triethylamine) as solvent A and CH3OH as solvent B, the gradient program was as follows: 55% A/45% B, 1mL/min, purity 96.74%.

The inhibitors i-j were prepared according to Scheme S3.

Scheme S3. Synthetic route of inhibitors i-j. Reagents and conditions: (i) Pd(OAc)2, BINAP, K2CO3, KI, toluene, 110 ˚C reflux，20 h; (ii) NaOH, H2O/MeOH, HCl, rt, 12 h; (iii) SOCl2,70 ˚C，5 h，then TEA, THF, rt，12 h.

ethyl 3-methoxy-4-((pyridin-3-ylmethyl)amino)benzoate (9)

The synthesis of intermediate 9 is the starting material 3-(chloromethyl)pyridine hydrochloride (98.02 mg, 768.37 μmol), 4-amino-3-methoxybenzoate ethyl (100 mg, 512.25 μmol), Palladium(II) acetate (28.75 mg, 128.06 μmol), BINAP (79.74 mg, 128.06 μmol), potassium carbonate (176.99 mg, 1.28 mmol) and potassium iodide (102.04 mg, 614.70 μmol) were dissolved in 15 mL of toluene, nitrogen protection. The reaction was stirred and refluxed in an oil bath at 110 ^o^C, and the reaction time was 20 h. After the reaction, the filtrate was filtered through Celite, and the organic solvent was evaporated. The crude product was separated and purified to obtain 45.58 mg of a yellow solid, with a yield of 31.07%. Melting point: 123.6-124.3℃. 1H NMR (400 MHz, DMSO-*d_6_*) δ 8.56 (s, 1H), 8.43 (d, J = 4.2 Hz, 1H), 7.71 (d, J = 8.0 Hz, 1H), 7.46–7.24 (m, 3H) , 6.58 (t, J = 6.2 Hz, 1H), 6.49 (d, J = 8.2 Hz, 1H), 4.44 (d, J = 6.0 Hz, 2H), 4.31–4.15 (m, 2H), 3.86 (s, 3H), 1.25 (d, J = 7.6 Hz, 3H). ESI-MS: m/z [M + H]+ cacd for C16H19N2O3+ 287.1, found 287.1.

3-methoxy-4-((pyridin-3-ylmethyl)amino)benzoic acid (10)

The synthesis of intermediate 10 is based on intermediate 9 (50 mg, 174.62 μmol) as the raw material, followed by the synthesis of intermediate 3b, to obtain 30.26 mg of a white powdery solid with a yield of 61.8%. Melting point: 263.4-264.5 ^o^C. 1H NMR (400 MHz, DMSO-*d_6_*) δ 8.54 (d, J = 16.2 Hz, 1H), 8.43 (d, J = 8.8 Hz, 1H), 7.72 (d, J = 7.1 Hz, 1H), 7.48–7.18 (m, 3H), 6.33 (d, J = 8.1 Hz, 1H), 5.75 (d, J = 12.2 Hz, 1H), 4.37 (d, J = 6.2 Hz, 2H), 3.78 (d, J = 21.4 Hz , 3H). ESI-MS: m/z [M + H]+ cacd for C14H15N2O3+ 259.1, found 259.2.

(3-methoxy-4-((pyridin-3-ylmethyl)amino)phenyl)(8-oxa-2-azaspiro[4.5]decan-2-yl)methanone (i)

The target compound i was synthesized with intermediate 10 (50 mg, 178.41 μmol) as the raw material, 5 mL of thionyl chloride as the solvent, and the reaction was refluxed in an oil bath at 70°C for 5 h. After the reaction is completed, the thionyl chloride is distilled off, put into a vacuum oil pump to remove the thionyl chloride as much as possible, and the raw material becomes acid chloride. Dissolve 8-oxa-2-azaspiro[4.5]decane (37.79 mg, 267.61 μmol) and triethylamine (74.19 μl, 535.22 μmol) in 5 mL tetrahydrofuran, dissolve the acid chloride in 5 mL tetrahydrofuran, and drop at room temperature. Add to the tetrahydrofuran solution of spiroane and stir at room temperature for 12 h. After the reaction, the organic solvent was distilled off, 10 mL of distilled water was added, and the mixture was extracted three times with dichloromethane. The organic phases were combined and washed three times with distilled water, and washed three times with saturated sodium chloride solution. The organic phase was retained and dried with anhydrous sodium sulfate for 2 h. The anhydrous sodium sulfate was filtered off, the filtrate was collected, and the organic solvent was evaporated. The crude product was separated and purified to obtain a pale powder solid 14.37 mg, with a yield of 21.1%. Melting point: 128.2-129.6 ^o^C. 1H NMR (400 MHz, DMSO-*d_6_*) δ 8.46 (s, 1H), 8.35 (t, J = 15.3 Hz, 1H), 7.65 (d, J = 7.4 Hz, 1H), 7.50 (s, 1H), 7.32–7.24 (m, 1H), 7.06 (d, J = 20.7 Hz, 1H), 6.96 (s, 1H), 5.68 (dd, J = 26.7, 19.6 Hz, 1H), 4.65 – 4.45 (m, 2H), 3.76 (s, 3H), 3.49 (dd, J = 20.4, 16.2 Hz, 6H), 1.76 (d, J = 7.4 Hz, 2H), 1.45 (d, J = 6.5 Hz, 4H), 1.23 (s, 2H ).13C NMR (101 MHz, DMSO-*d_6_*) δ 166.34, 149.98, 149.24, 148.37, 136.83, 136.81, 136.20, 135.44, 135.41, 123.75, 121.76, 120.87, 65.16, 64.69, 64.35, 56.60, 46.96, 40.96, 40.96 , 40.22, 40.01, 39.80, 39.59, 39.38, 37.07, 36.68, 32.45.. ESI-HRMS: m/z [M+H]+ cacd for C22H28N3O3+ 382.2131, found 382.2131. HPLC analysis :Retention time = 13.850 min, C8 reversed-phase column (250 mm × 4.6 mm, 5 μm, Phenomenex), H2O (containing 0.1% triethylamine) as solvent A and CH3OH as solvent B, the gradient program was as follows: 55% A/45% B, 1mL/ min, purity 99.38%.

(3-methoxy-4-((pyridin-3-ylmethyl)amino)phenyl)(7-oxa-2-azaspiro[3.5]nonan-2-yl)methanone (j）

The synthesis of the target compound j is based on the intermediate 10 (50 mg, 178.41 μmol) and 7-oxa-2-azaspiro[3.5]nonane (34.04 mg, 267.61 μmol) as raw materials, according to the synthesis method of the target product i.18.36 mg of slightly yellow powdery solid was obtained, the yield was 28.0%, and the melting point was 136.4-136.9 ^o^C. 1H NMR (400 MHz, DMSO-*d_6_*) δ 8.45 (s, 1H), 8.38 (s, 1H), 7.63 (t, J = 15.0 Hz, 1H), 7.60–7.34 (m, 1H), 7.31–7.25 (m, 1H), 7.15 (d, J = 13.1 Hz, 1H), 7.12–7.02 (m, 1H), 5.84 (t, J = 7.2 Hz, 1H), 4.77–4.47 (m, 2H), 4.09 (d , J = 12.5 Hz, 2H), 3.86 (dt, J = 22.1, 16.7 Hz, 2H), 3.75 (d, J = 18.3 Hz, 3H), 3.52 (d, J = 14.1 Hz, 4H), 1.69 (d , J = 13.1 Hz, 4H).13C NMR (101 MHz, DMSO-*d_6_*)δ167.80, 149.78, 149.15, 148.37, 137.10, 136.86, 135.33, 123.84, 123.79, 122.40, 120.48, 110.66, 64.35, 63.43, 56.55, 46.79, 40.63, 40.43, 40.22, 40.01, 39.80, 39.59, 39.38, 35.93, 33.39, 29.50.ESI-HRMS: m/z [M + H]+ cacd for C21H26N3O3+368.1974, found 368.1974. HPLC analysis: Retention time = 1.511 min, C8 reversed-phase column (250 mm × 4.6 mm, 5 μm, Phenomenex), H2O (containing 0.1% triethylamine) as solvent A and CH3OH as solvent B, the gradient program was as follows: 55% A/45% B, 1mL/min, purity 99.73%.

# Experiment graphs

Figure S1. Cytotoxicity test results of vanin-1 small molecule inhibitor. Our inhibitors have less cytotoxicity and will not interfere with the experimental results due to cytotoxicity under the experimental conditions of cell bioluminescence imaging.

# NMR and MS spectra of the target compound

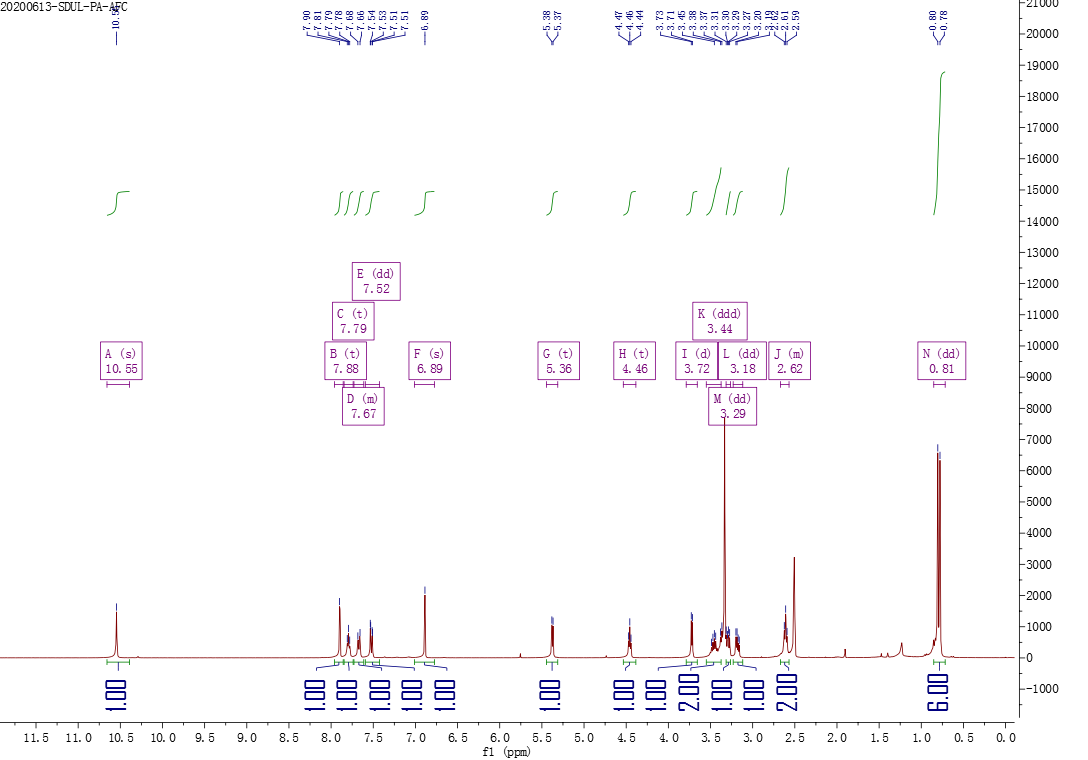


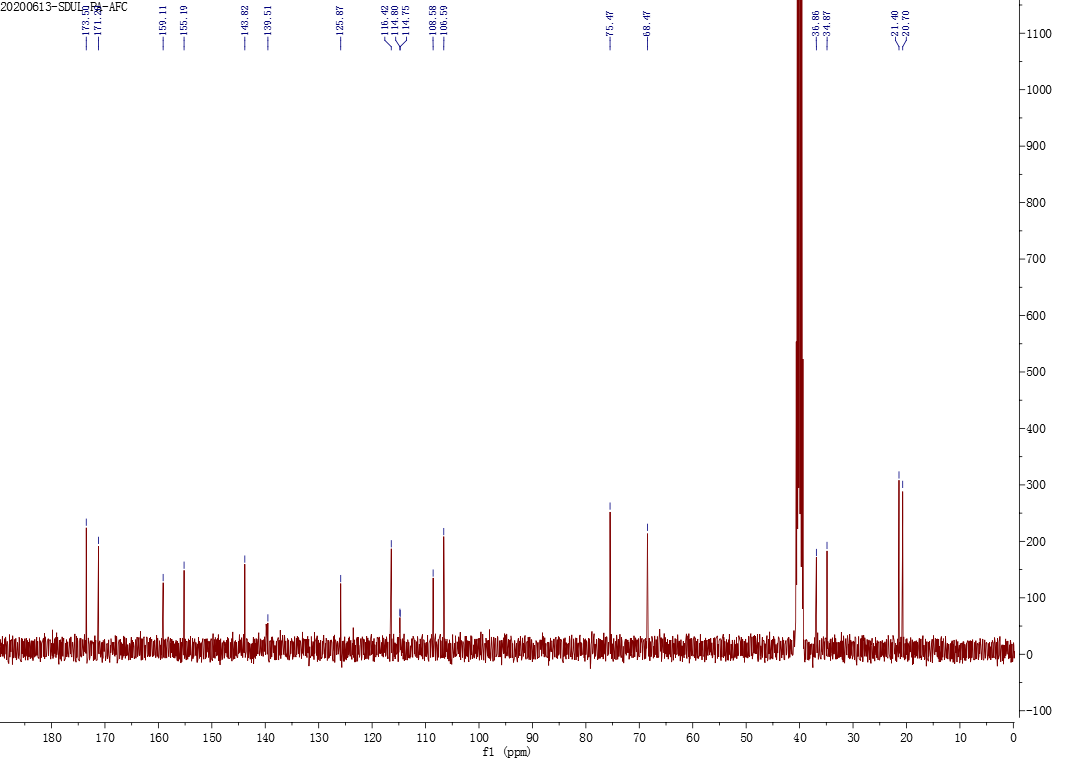


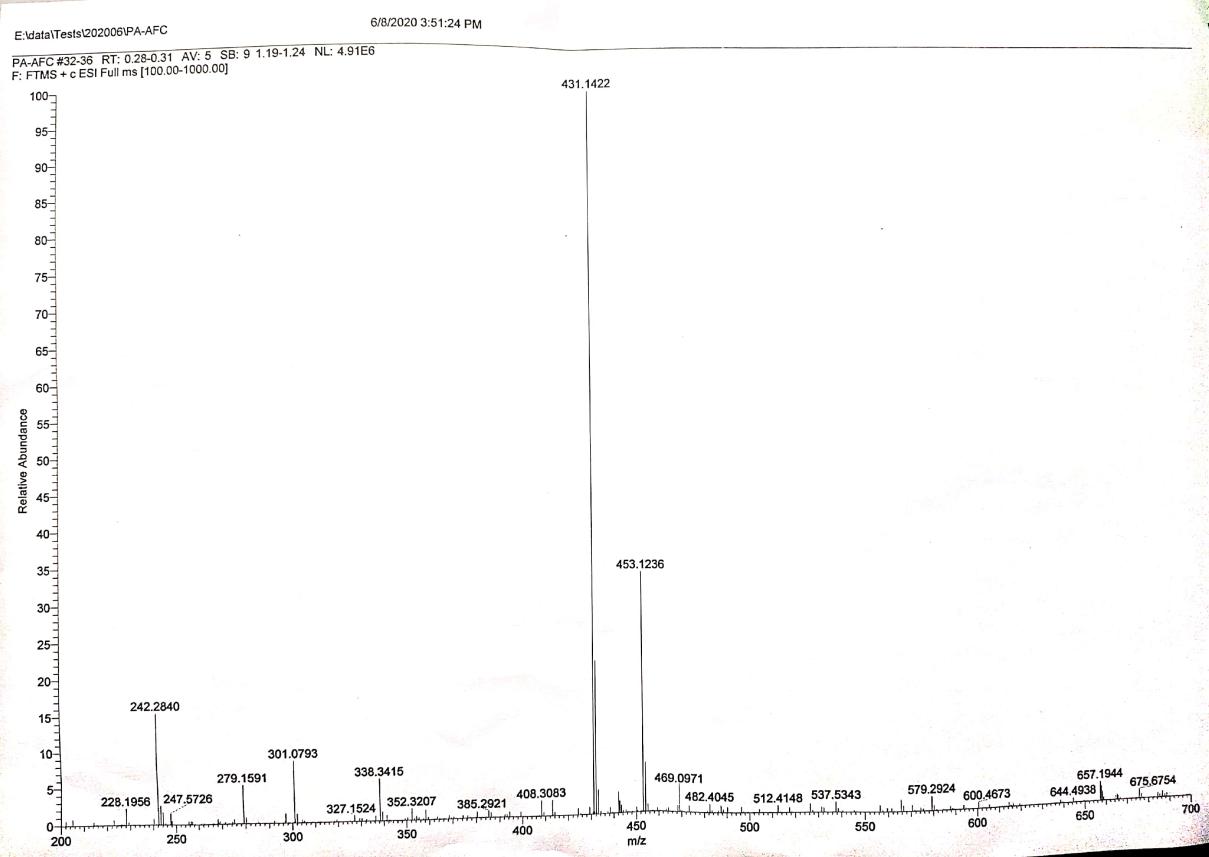

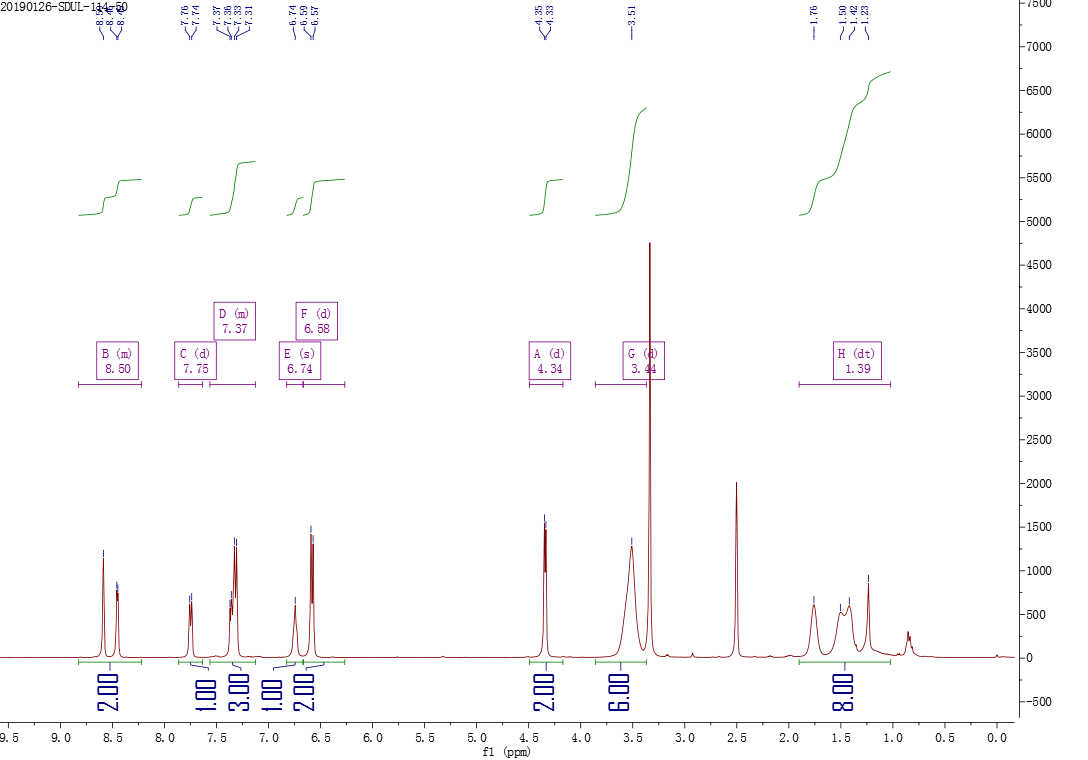


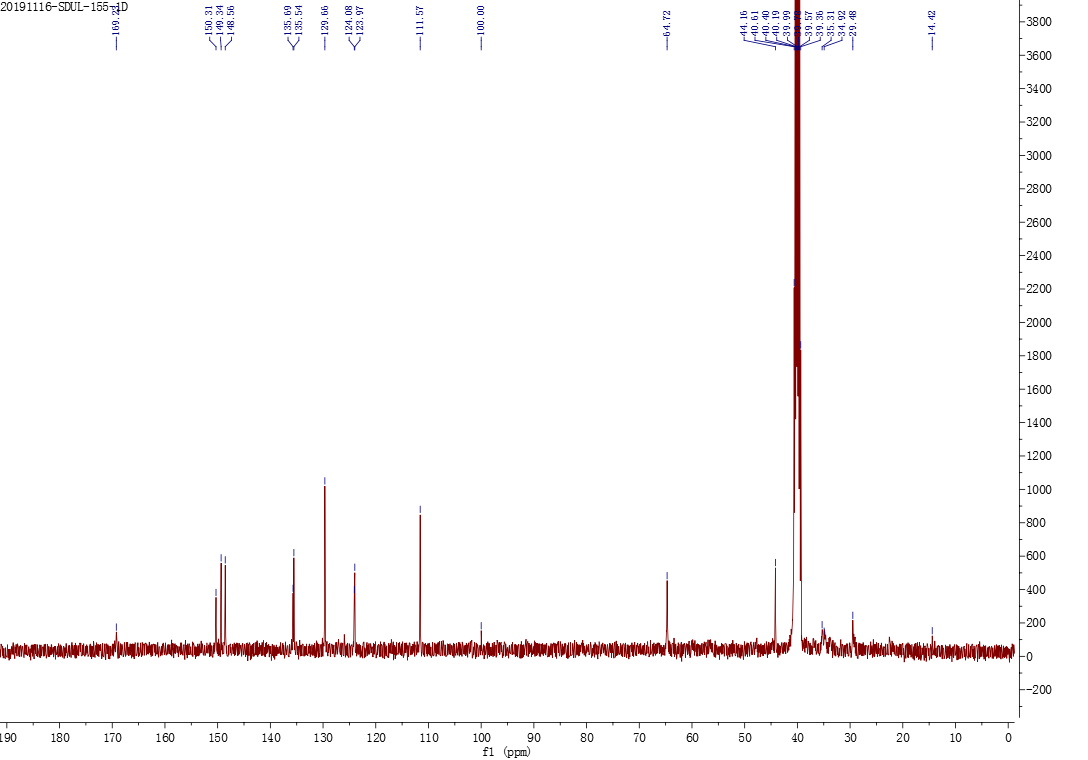


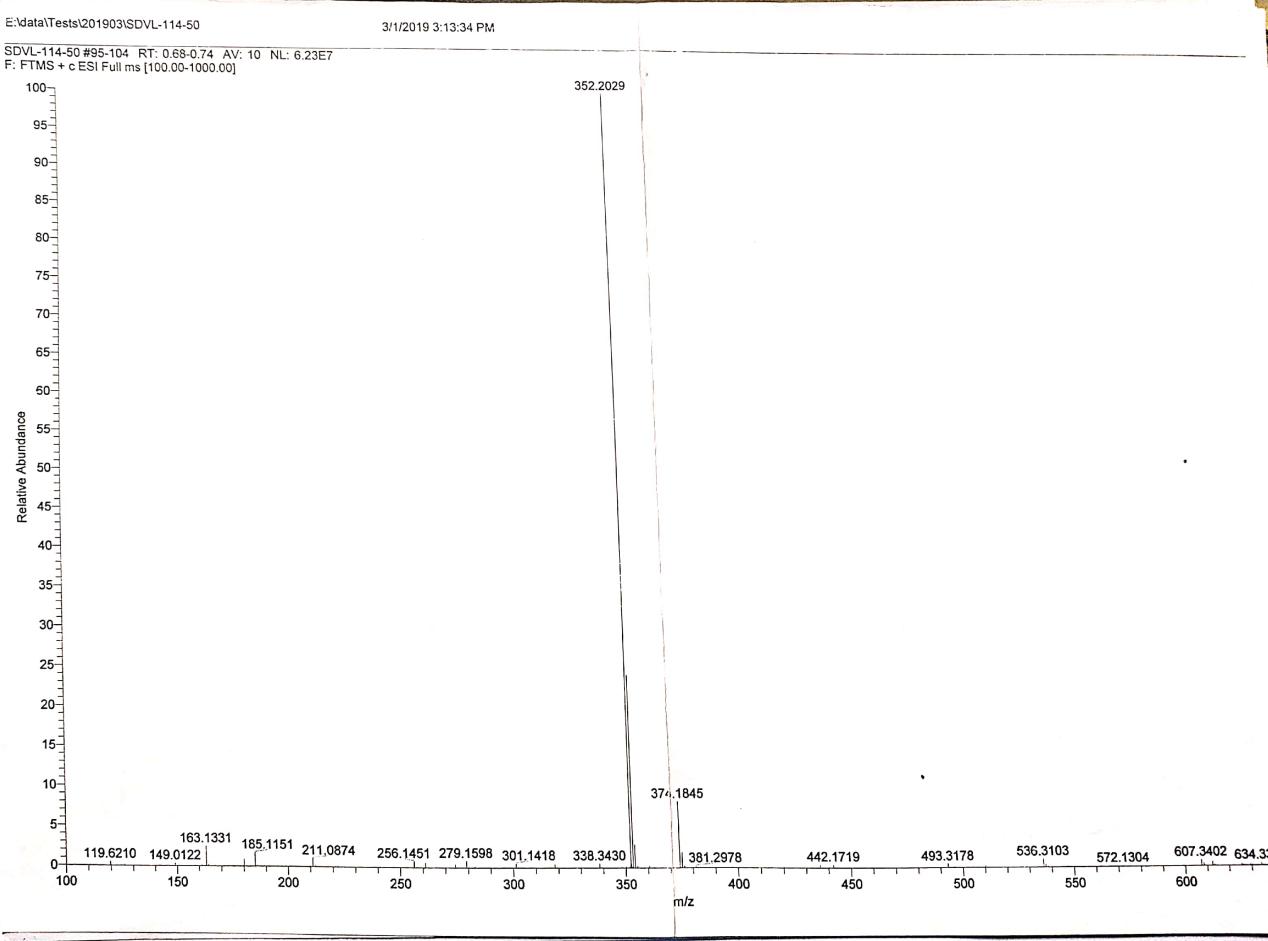

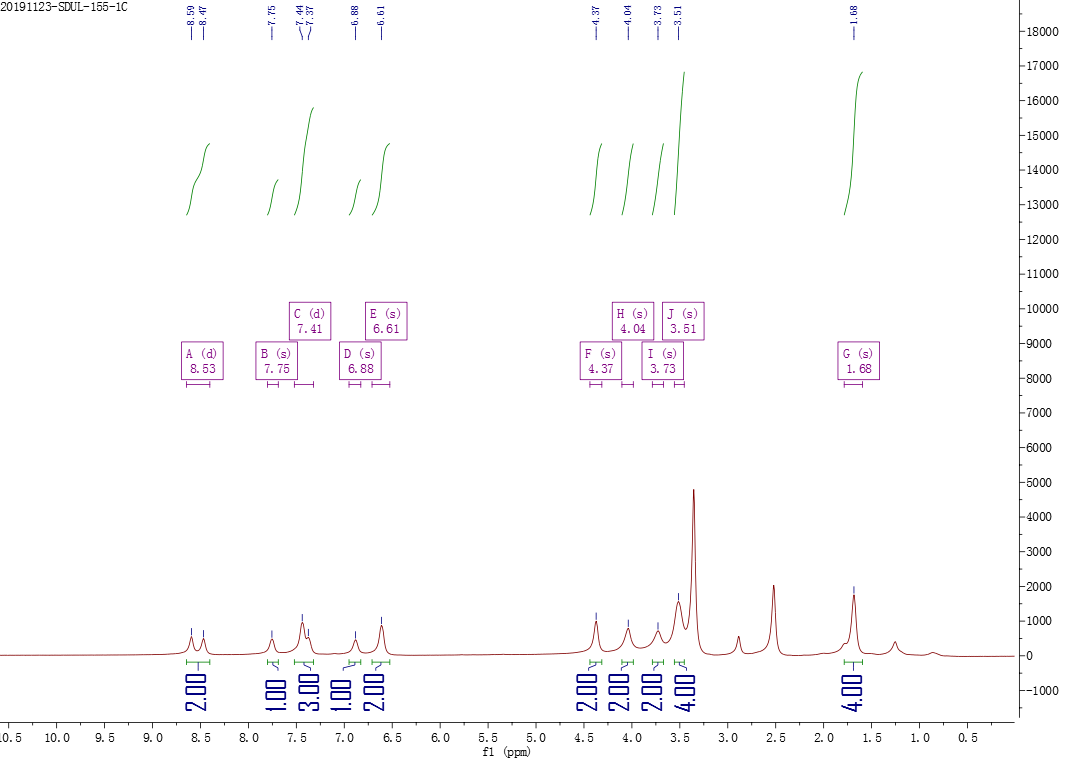


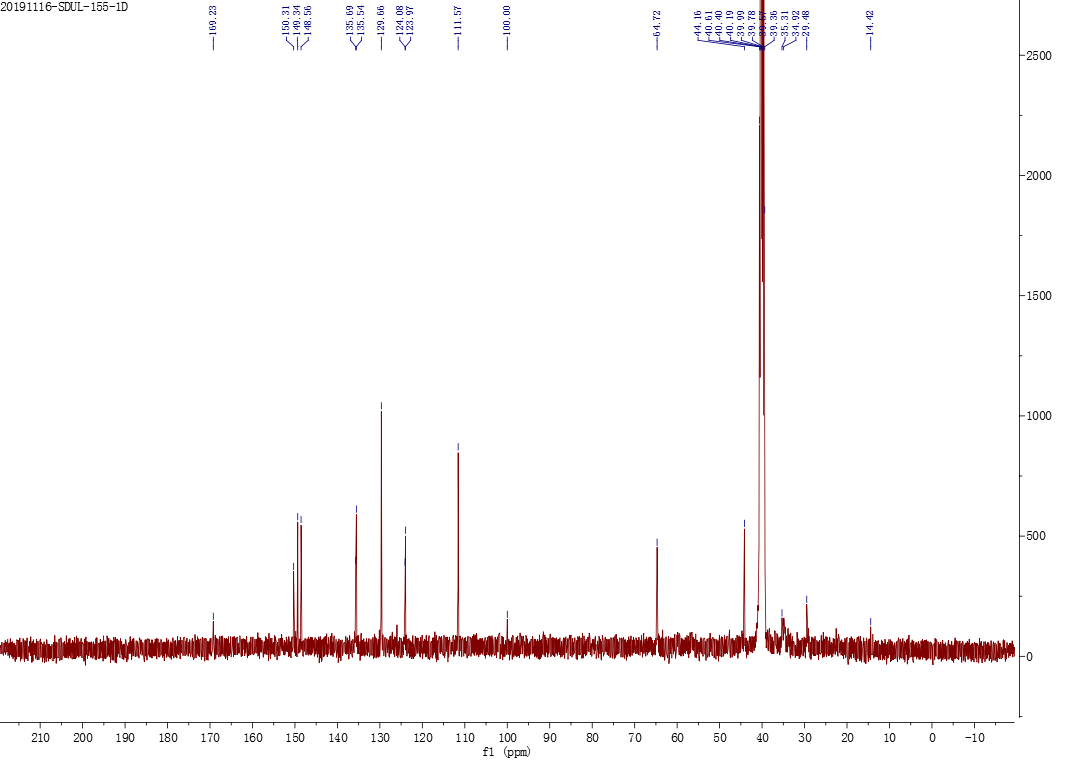


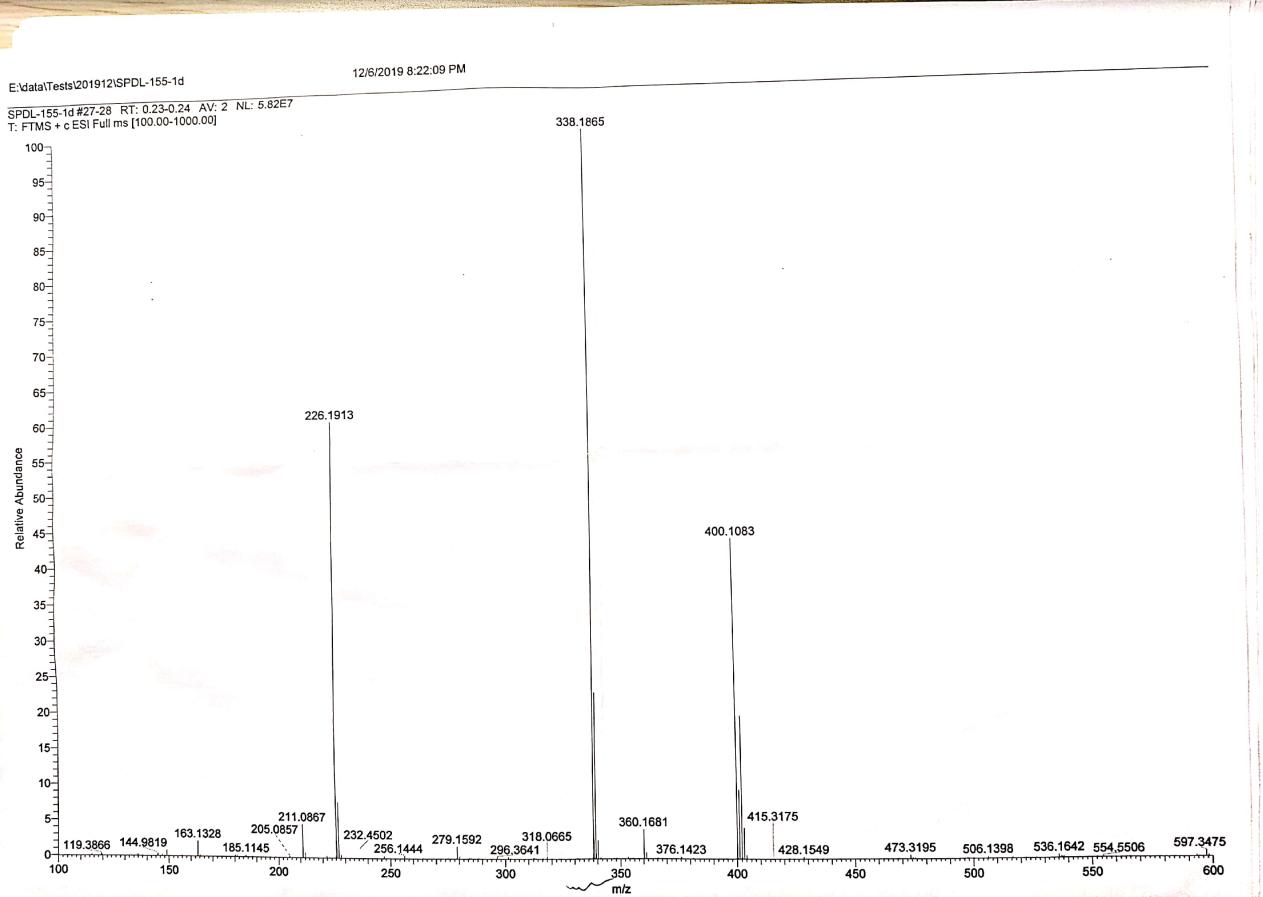

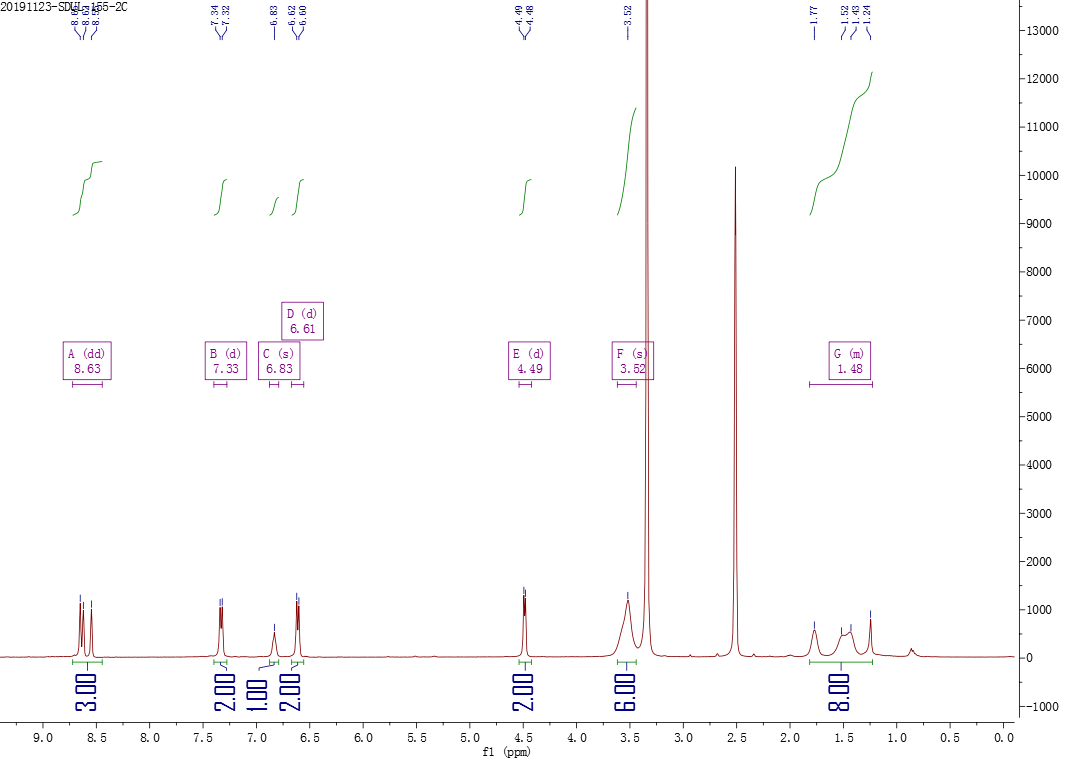


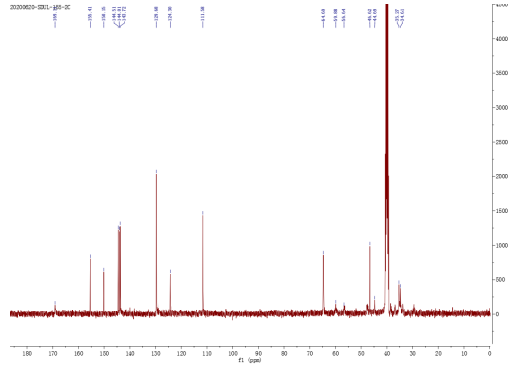

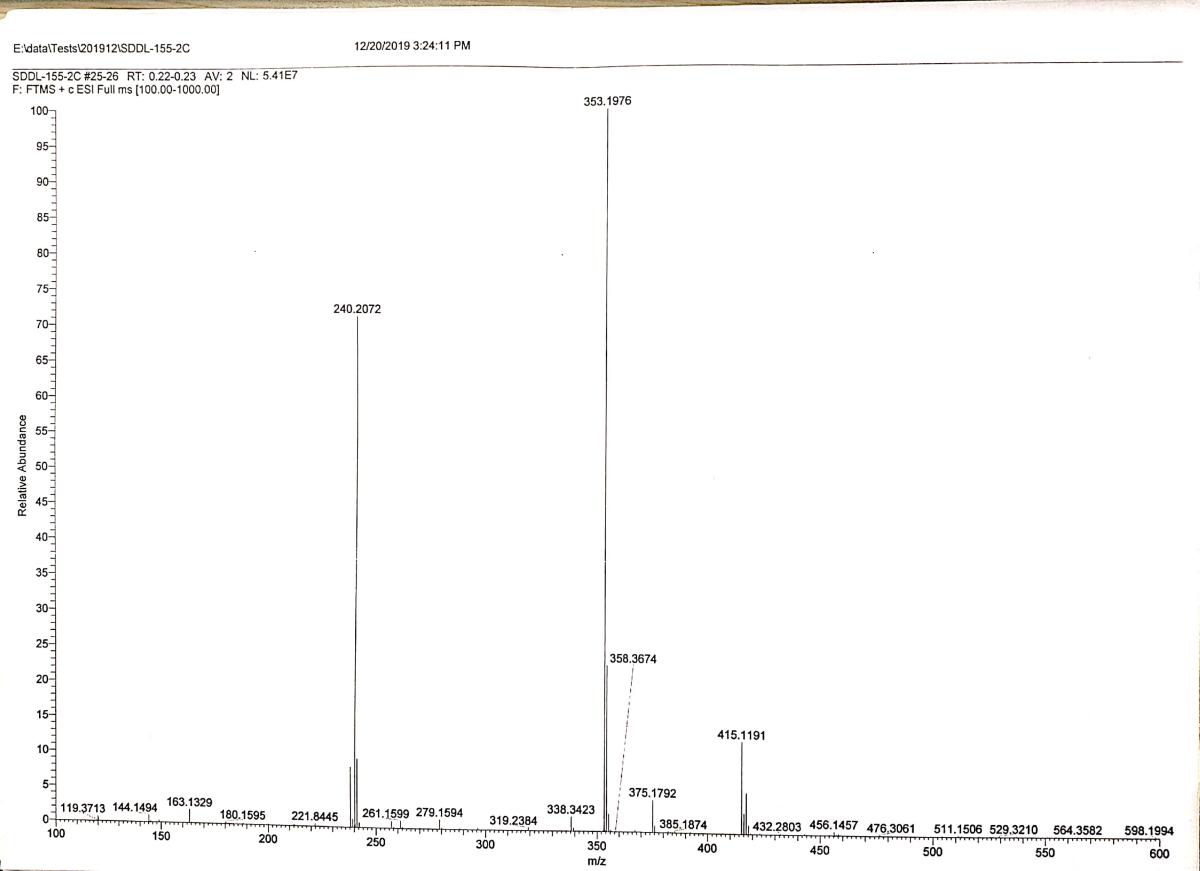


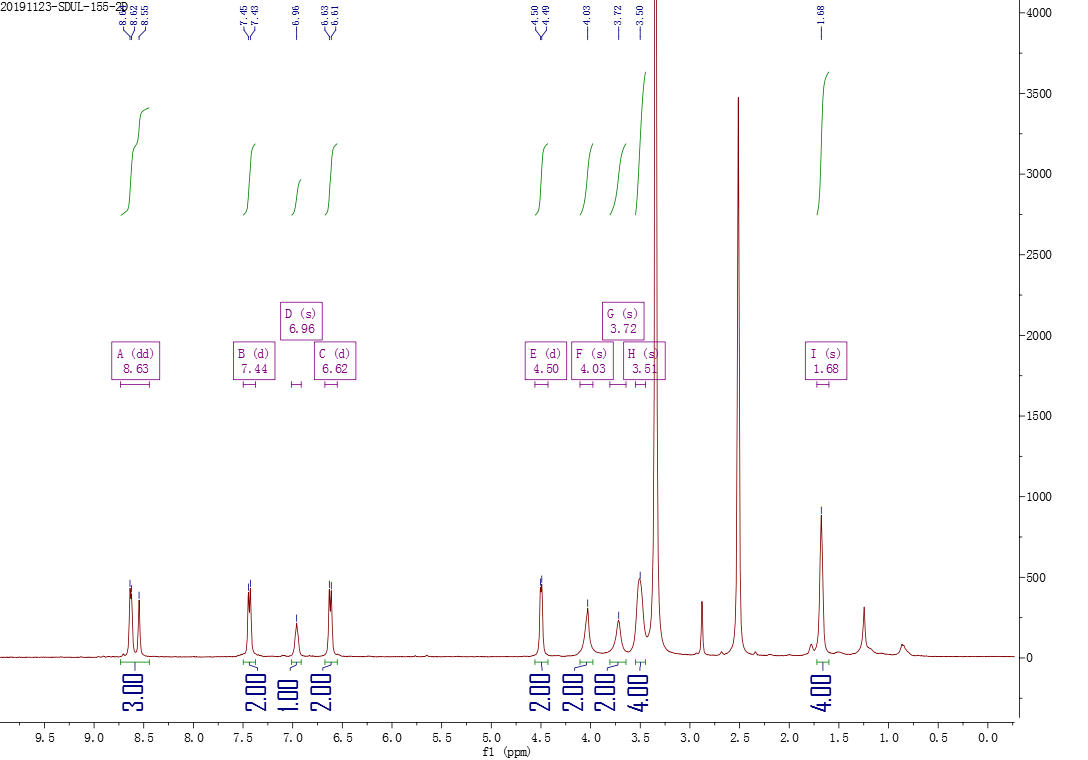


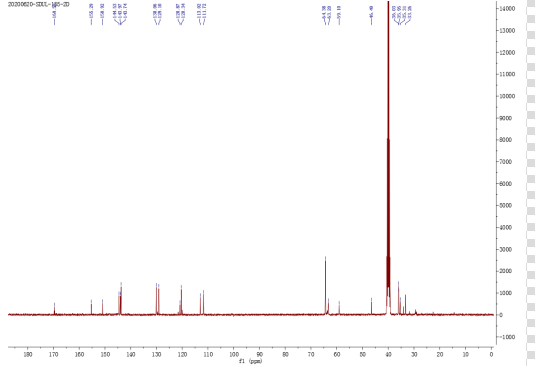


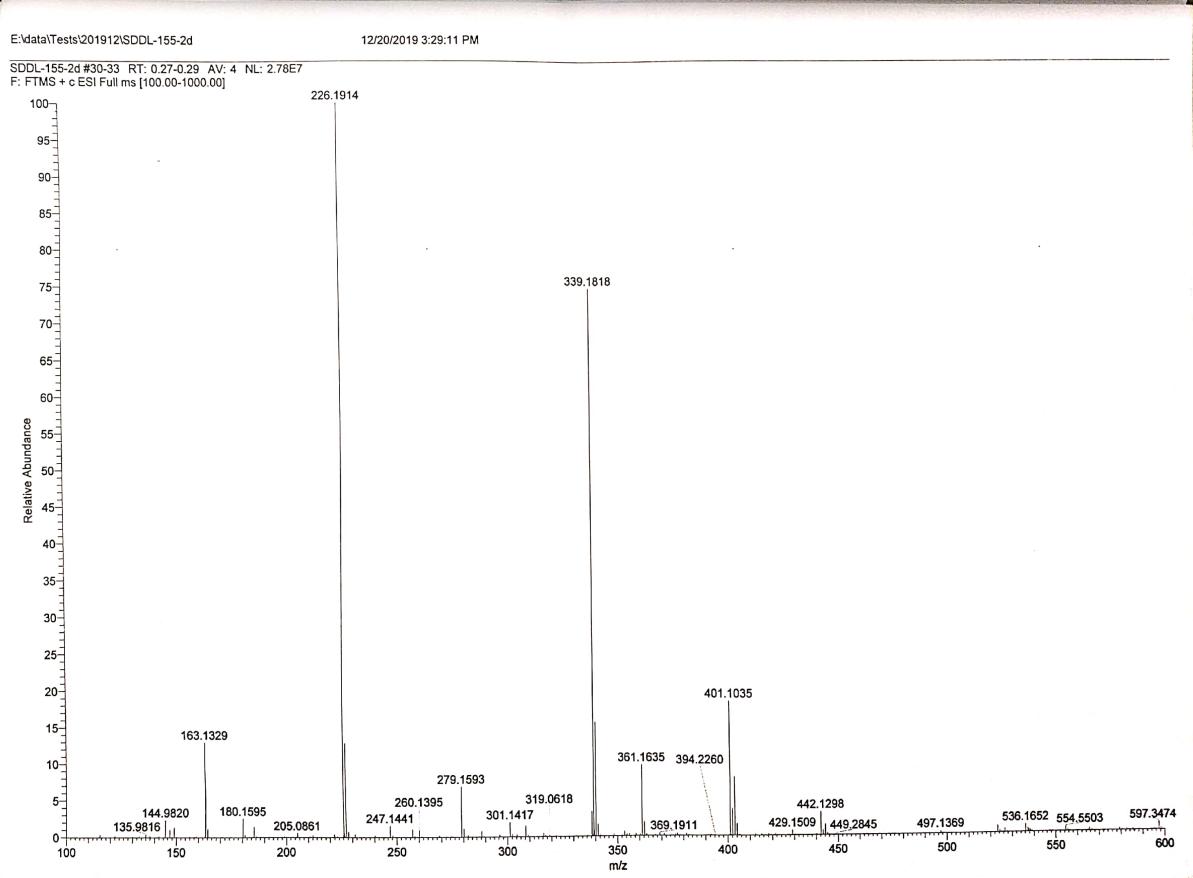

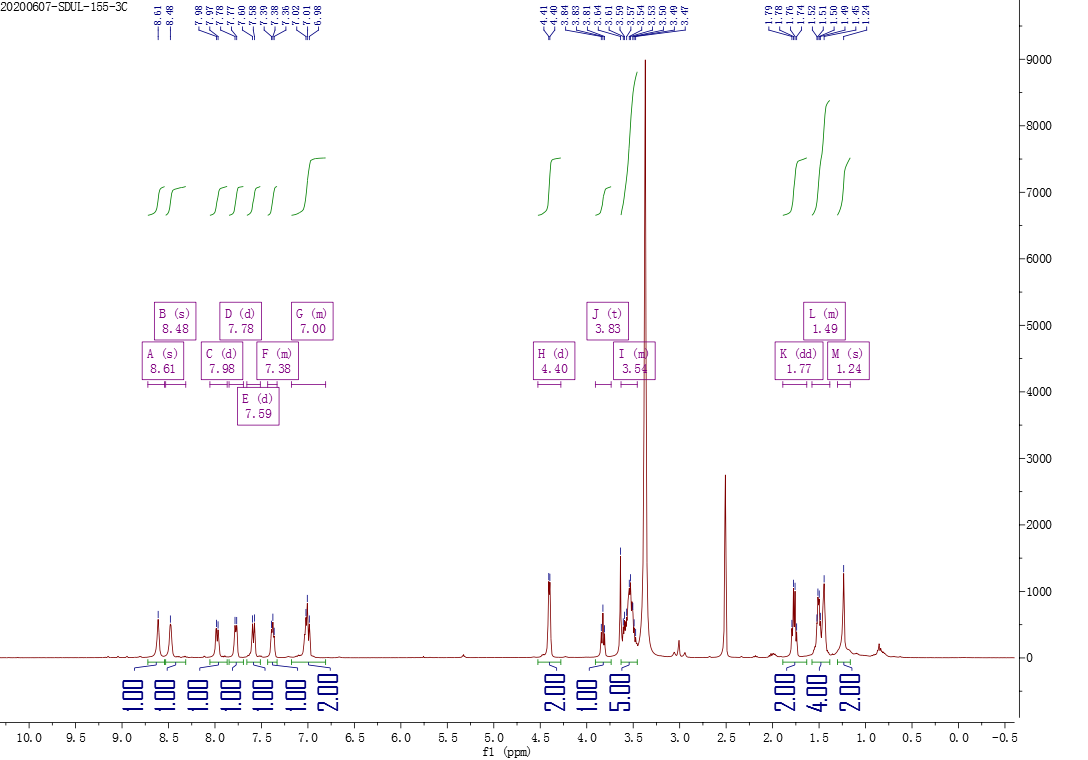


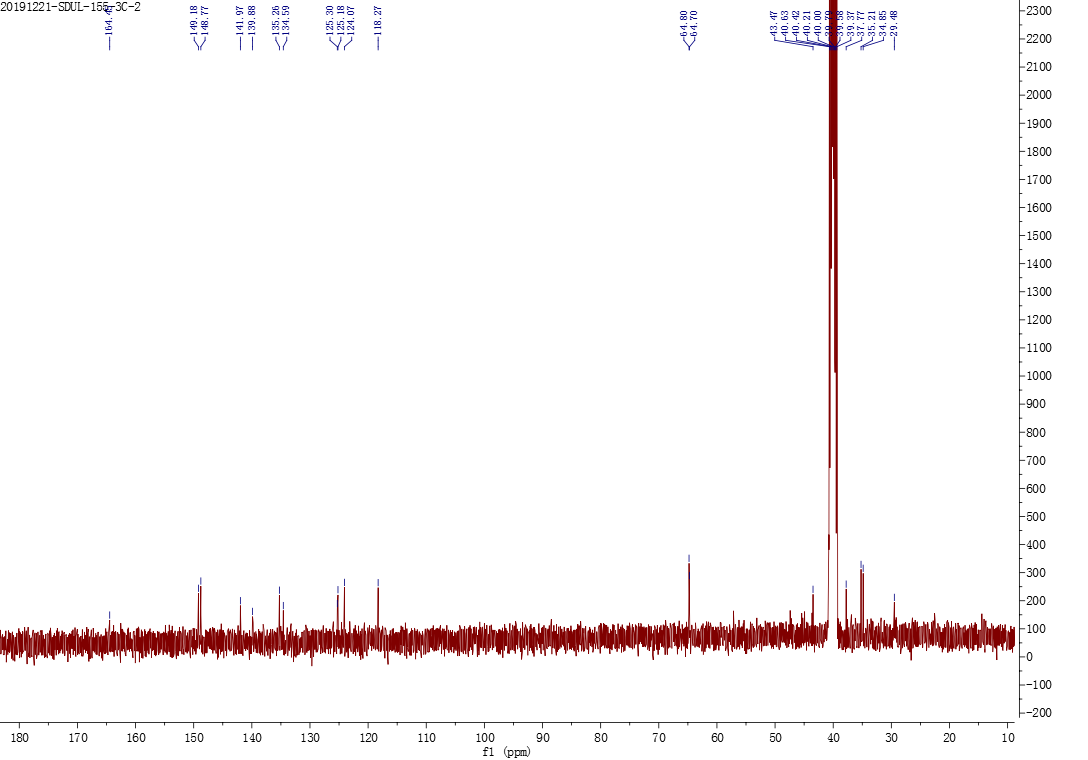


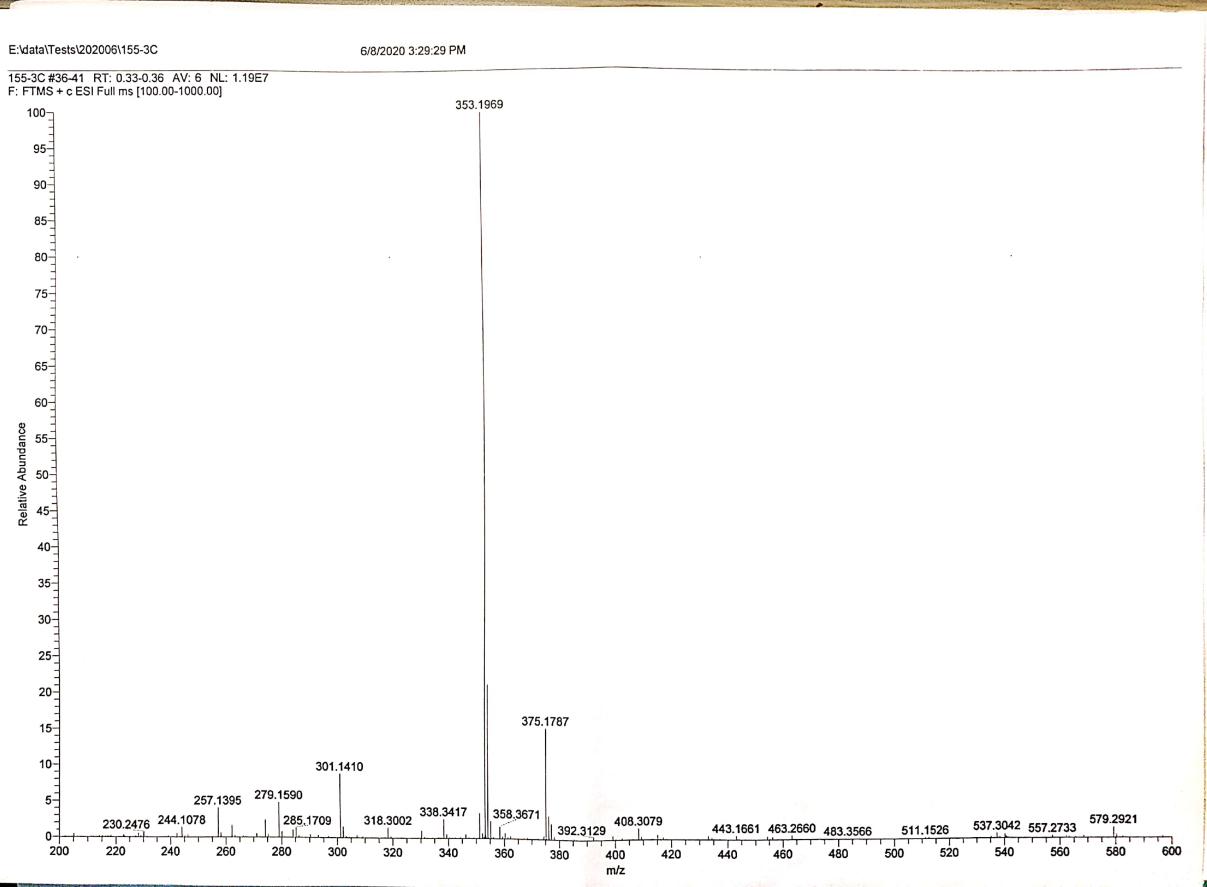

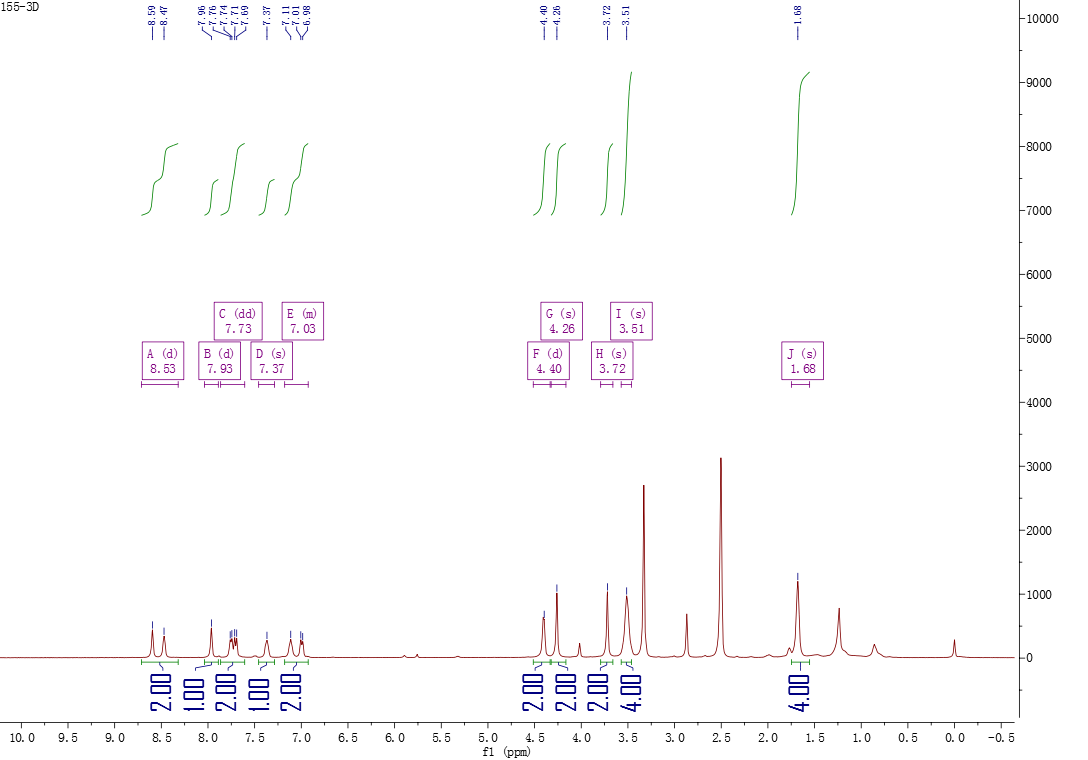


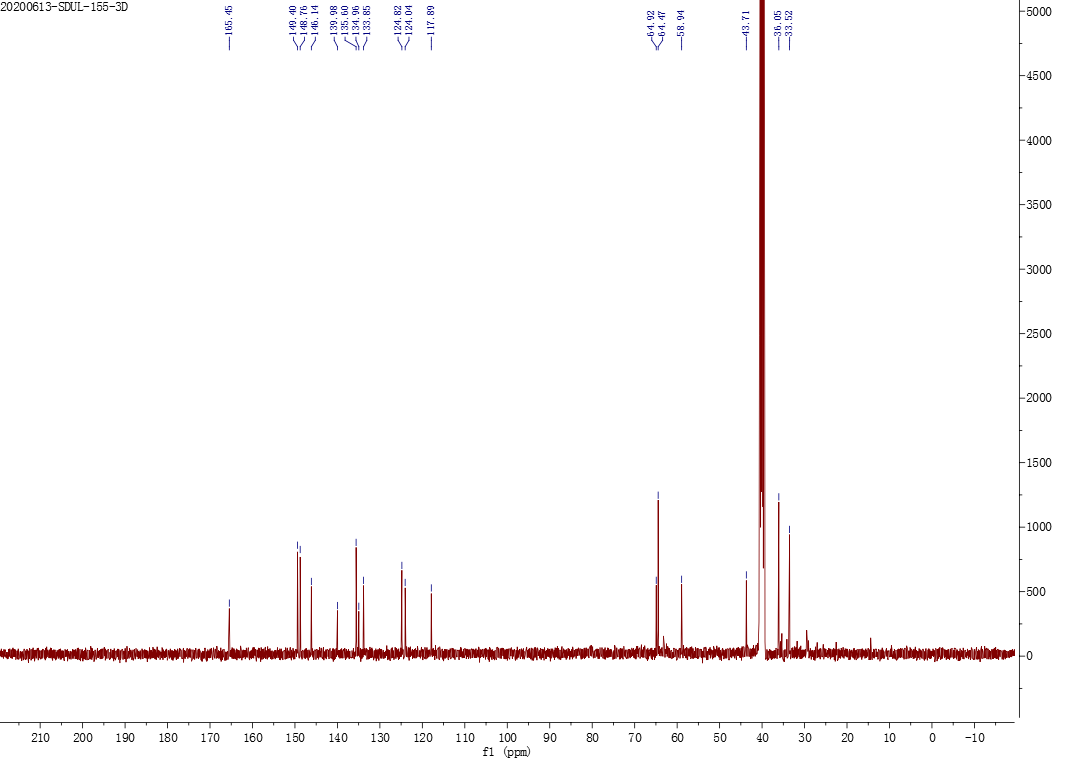


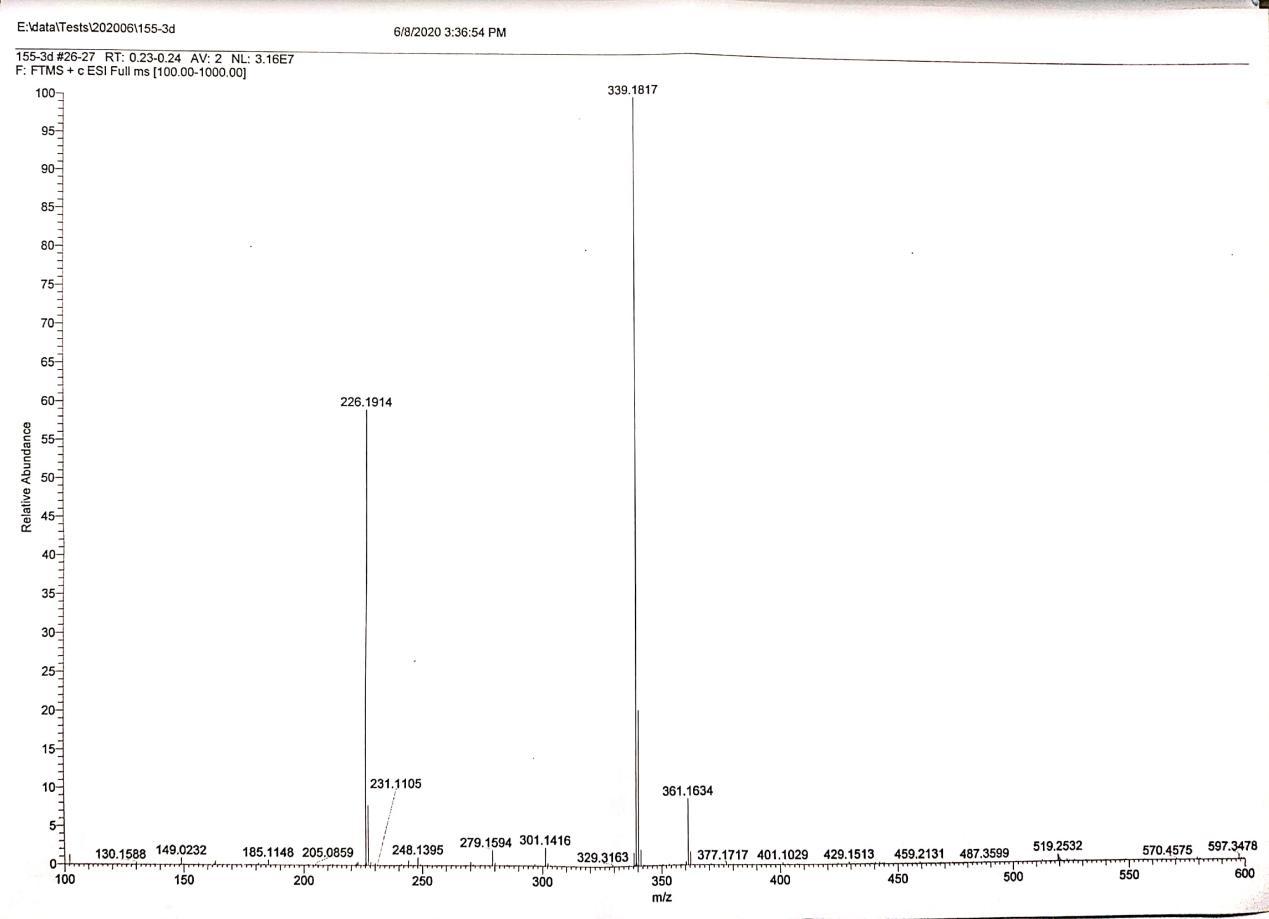


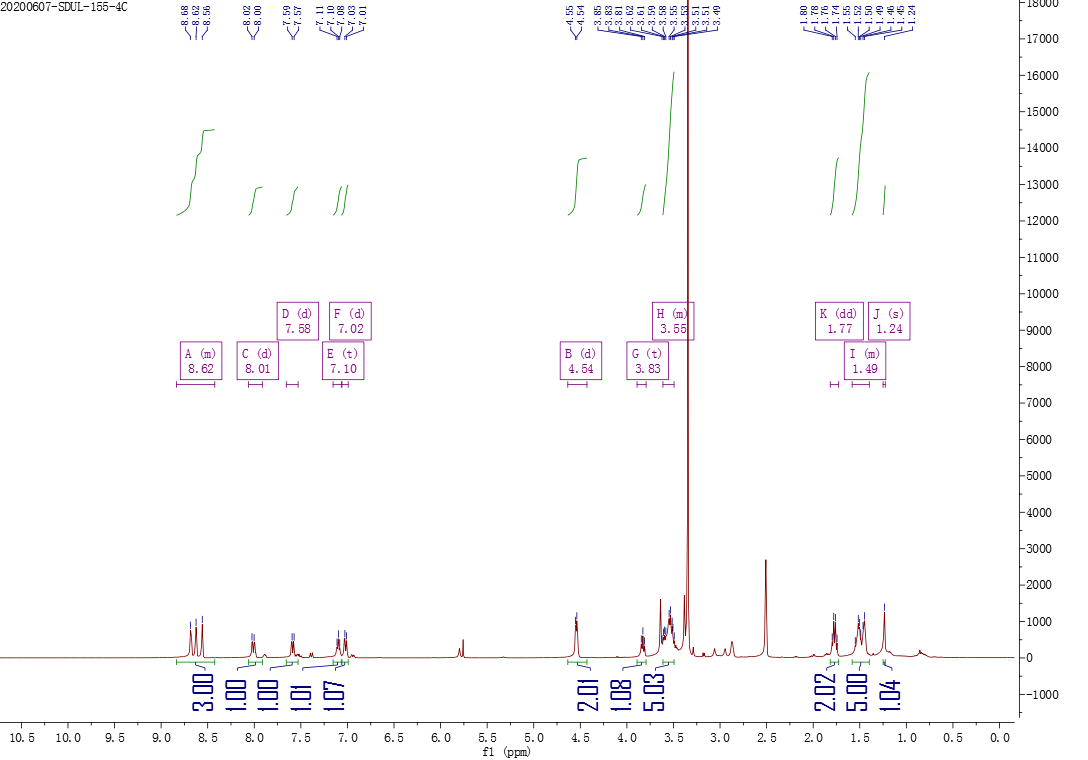


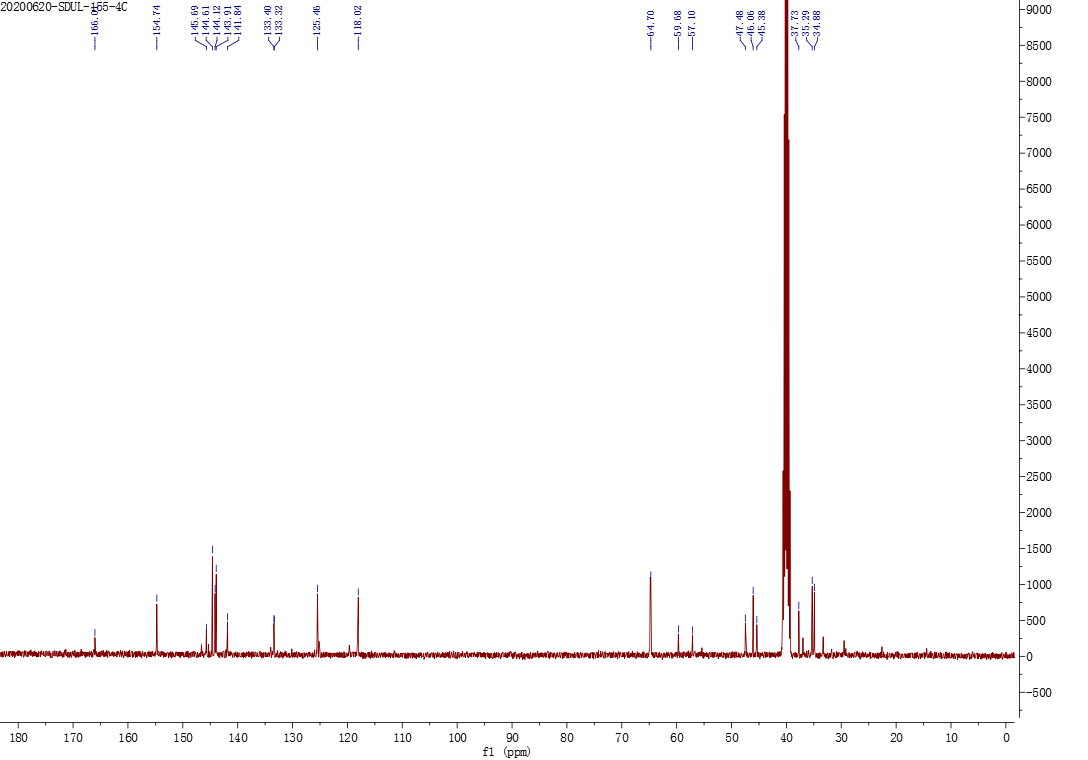


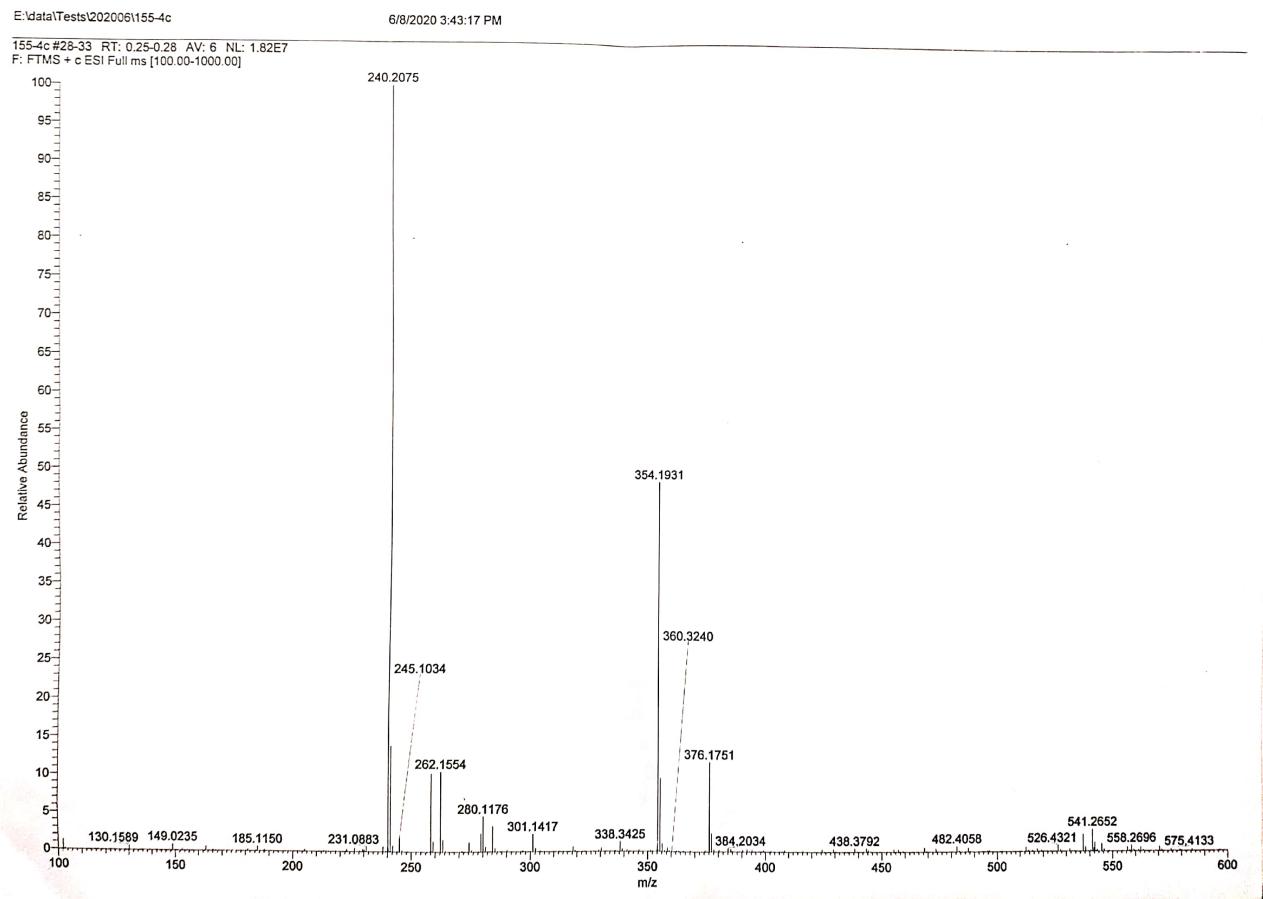


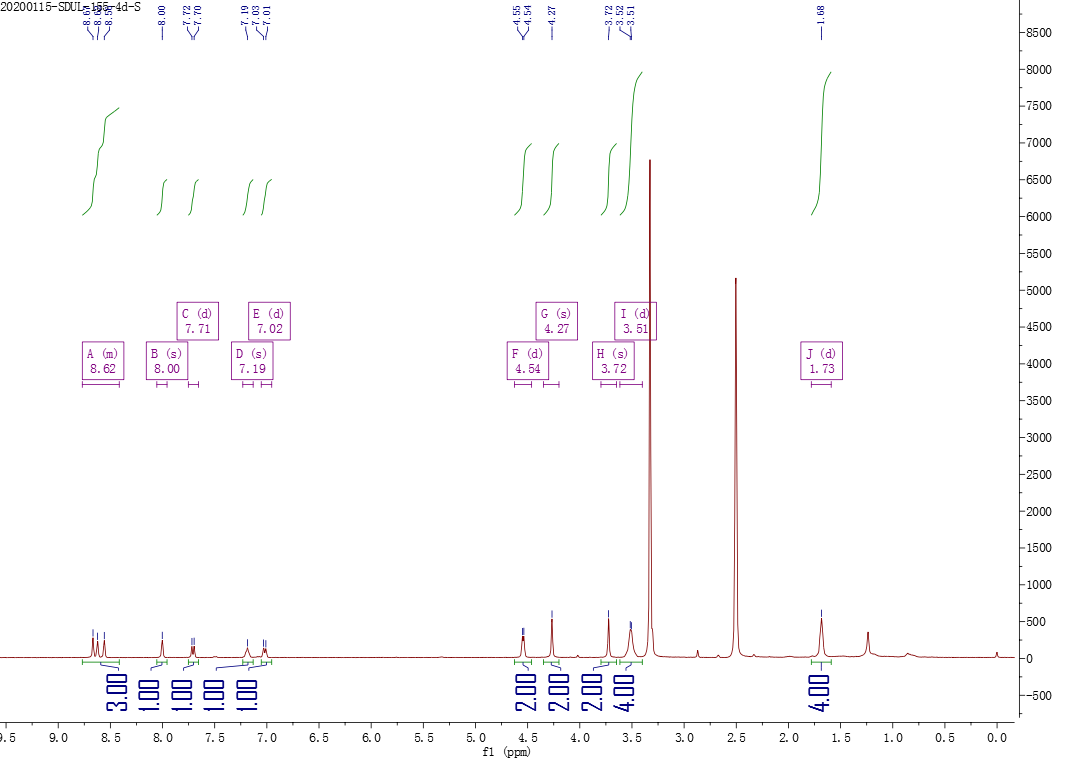


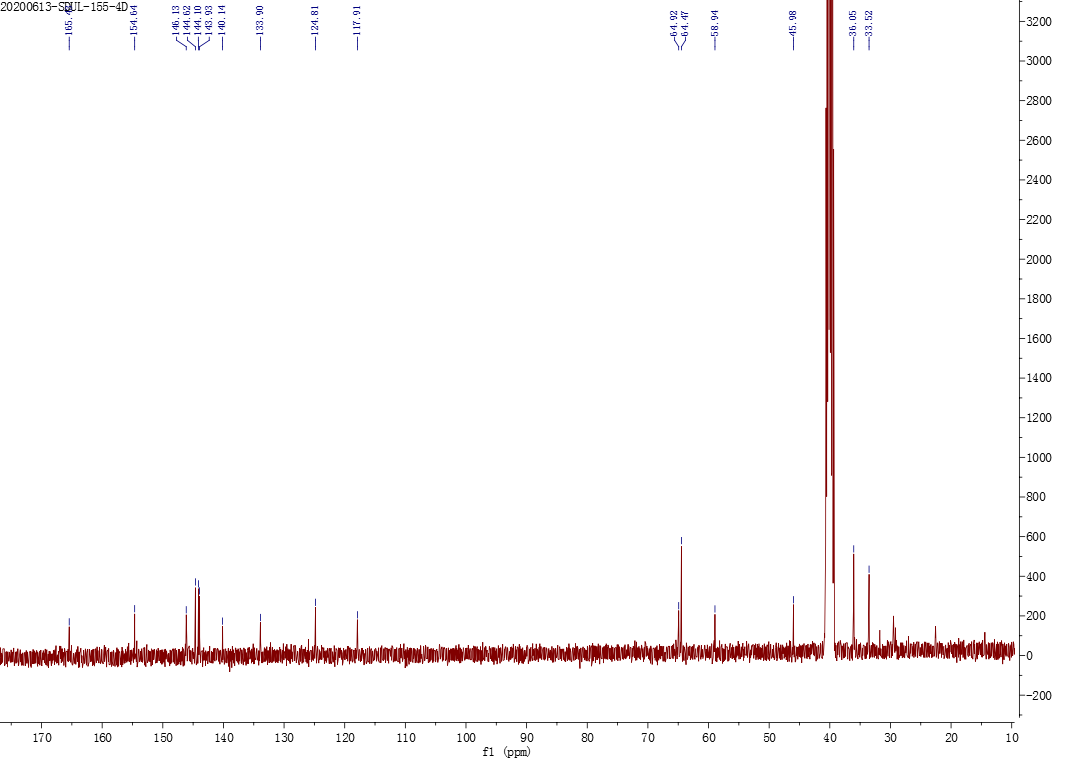


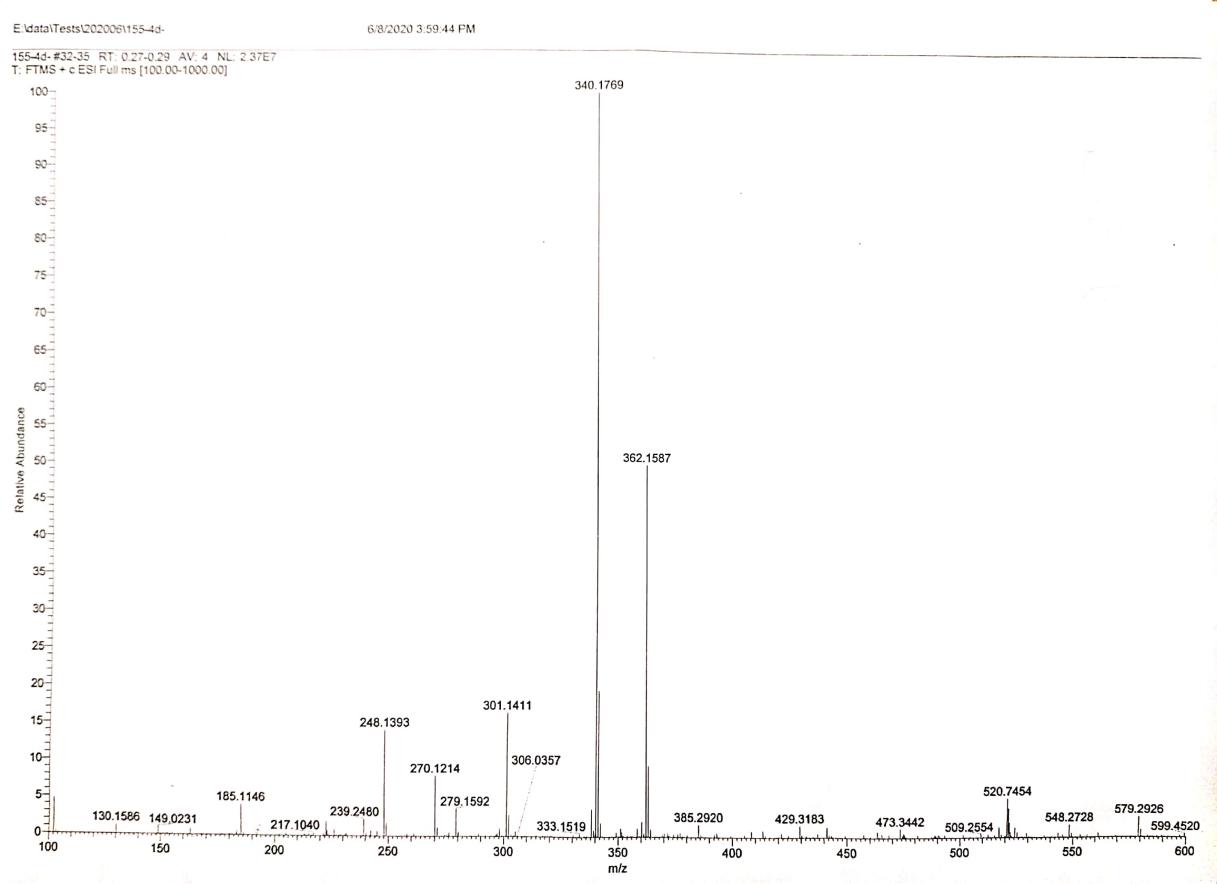

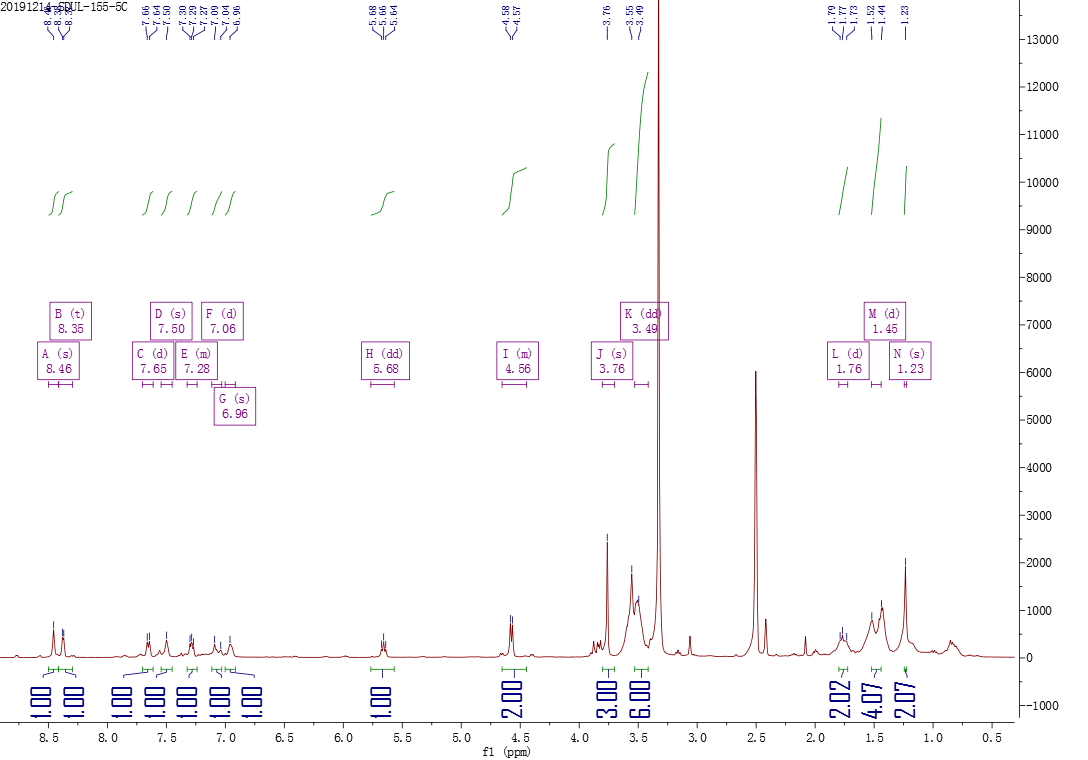


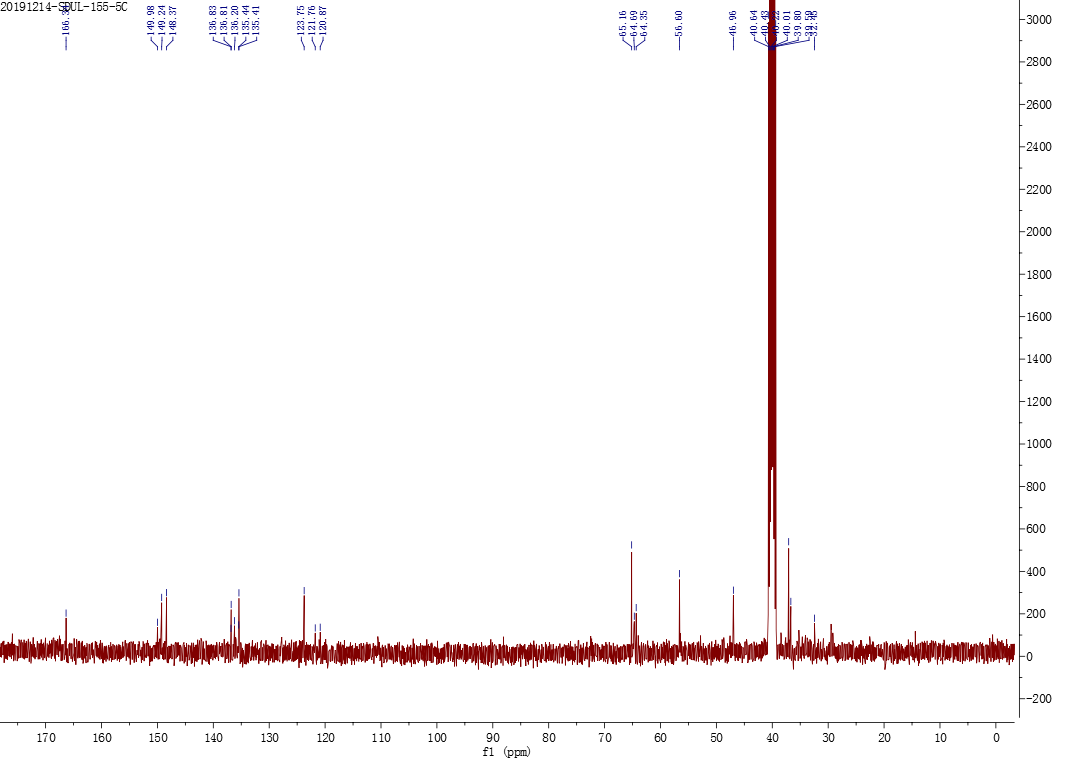

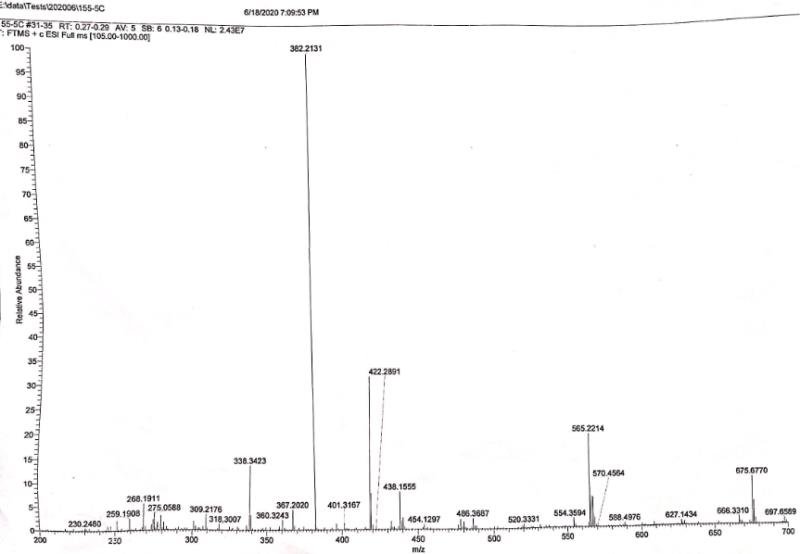

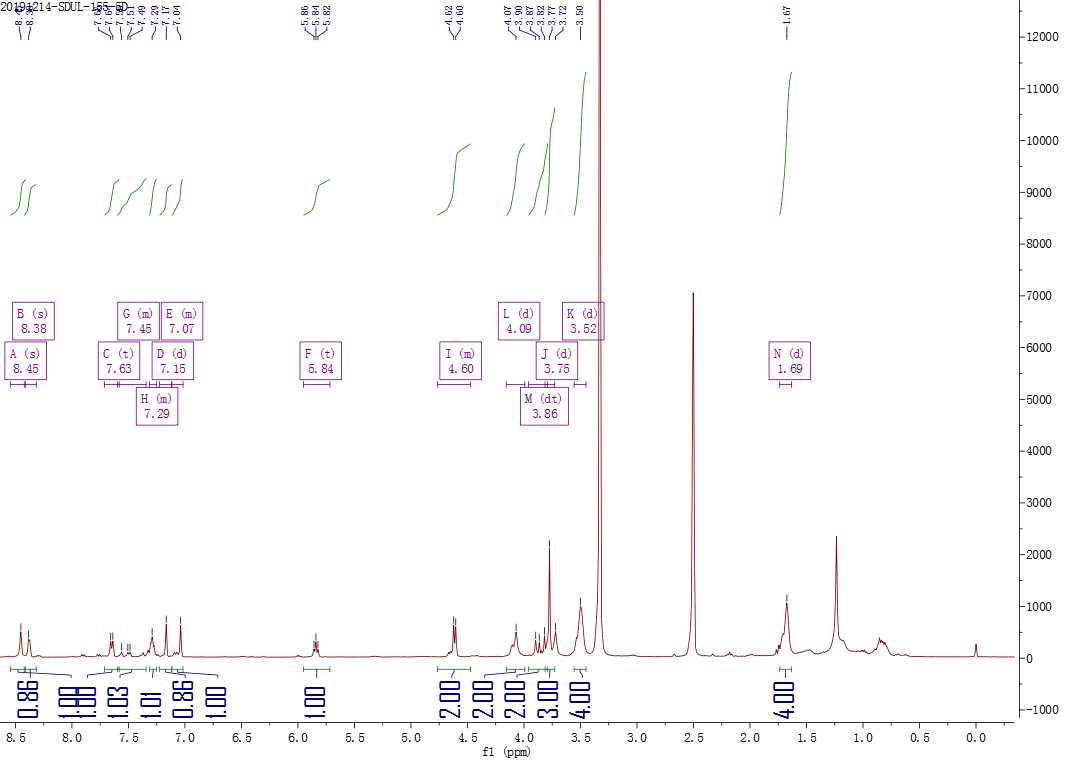


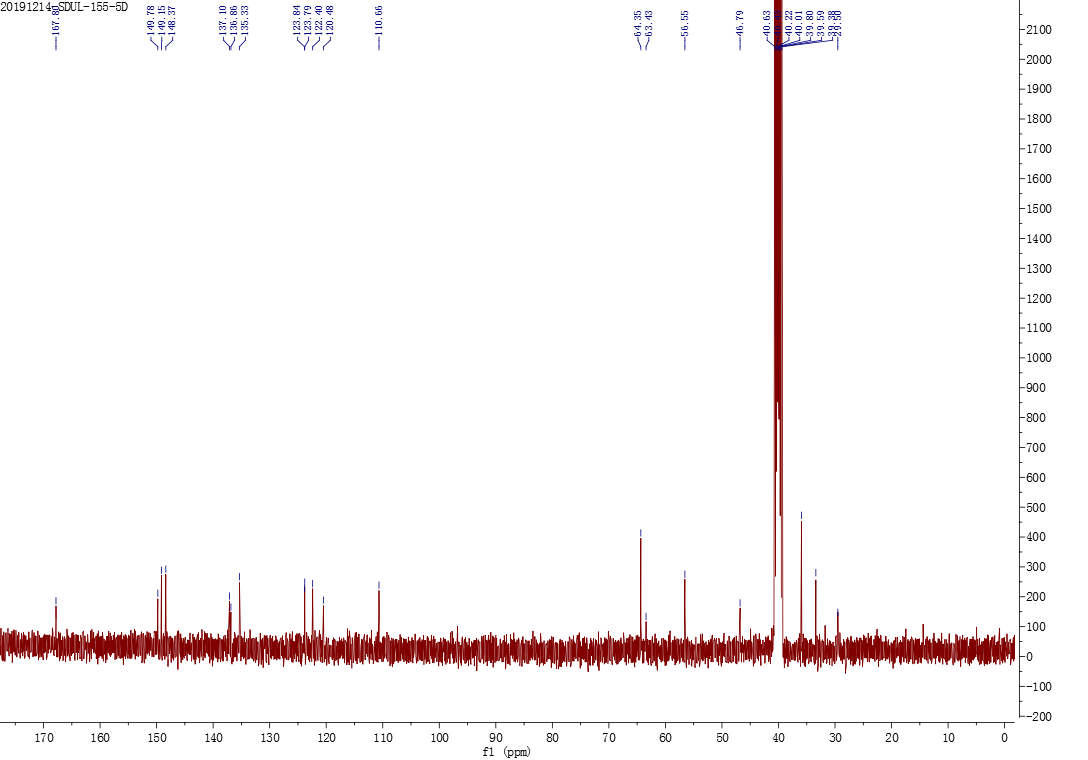


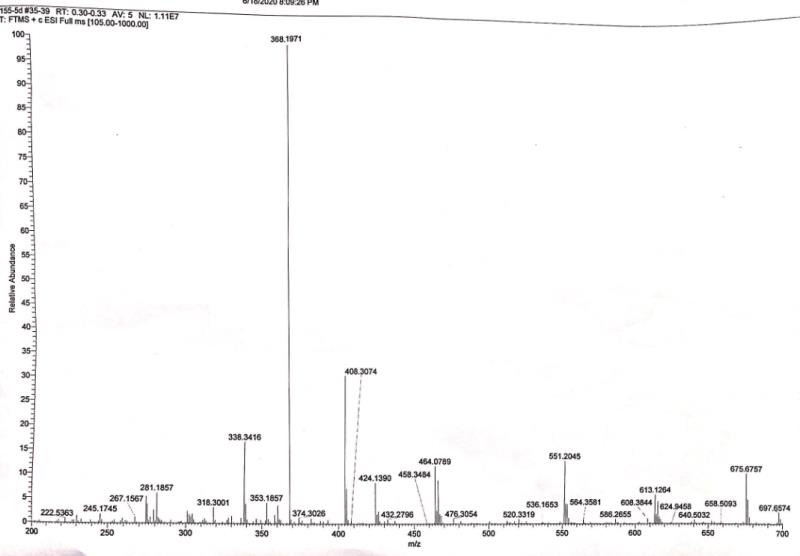


**
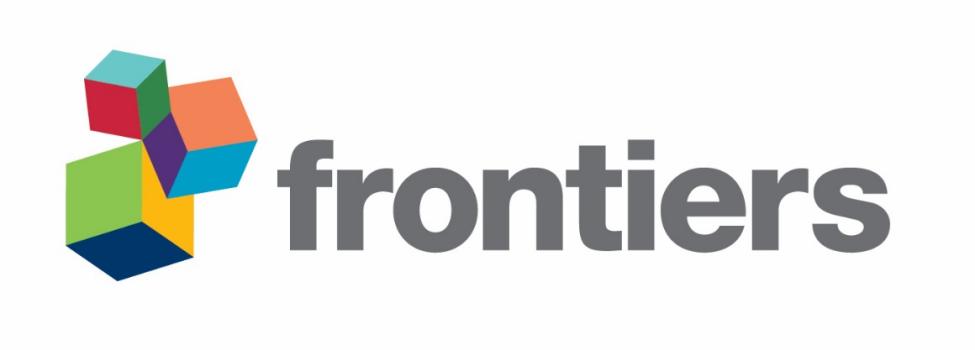
**
